# Management of SARS-CoV-2 Infection-Clinical Practice Guidelines of the Polish Association of Epidemiologists and Infectiologists, for 2025

**DOI:** 10.3390/jcm14072305

**Published:** 2025-03-27

**Authors:** Robert Flisiak, Jerzy Jaroszewicz, Dorota Kozielewicz, Ernest Kuchar, Miłosz Parczewski, Małgorzata Pawłowska, Anna Piekarska, Piotr Rzymski, Krzysztof Simon, Krzysztof Tomasiewicz, Dorota Zarębska-Michaluk

**Affiliations:** 1 Department of Infectious Diseases and Hepatology, Medical University in Białystok, 15-540 Białystok, Poland; 2 Department of Infectious Diseases and Hepatology, Medical University of Silesia, 40-635 Katowice, Poland; jerzy.jr@gmail.com; 3 Department of Infectious Diseases and Hepatology, Faculty of Medicine, Collegium Medicum Bydgoszcz, Nicolaus Copernicus University, 87-100 Toruń, Poland; d.kozielewicz@wsoz.pl (D.K.); mpawlowska@cm.umk.pl (M.P.); 4 Pediatric and Observation Department, Medical University of Warsaw, 02-091 Warszawa, Poland; ernest.kuchar@gmail.com; 5 Department of Infectious and Tropical Diseases and Acquired Immunodeficiency, Pomeranian Medical University, 70-204 Szczecin, Poland; milosz.parczewski@pum.edu.pl; 6 Department of Infectious Diseases and Hepatology, Medical University of Łódź, 90–419 Łódź, Poland; annapiekar@gmail.com; 7 Department of Environmental Medicine, Poznań University of Medical Sciences, 60-806 Poznań, Poland; rzymskipiotr@ump.edu.pl; 8 Department of Infectious Diseases and Hepatology, Medical University of Wrocław, 51-149 Wrocław, Poland; krzysimon@gmail.com; 9 Department of Infectious Diseases and Hepatology, Medical University of Lublin, 20-081 Lublin, Poland; tomaskdr@poczta.fm; 10 Department of Infectious Diseases and Allergology, Jan Kochanowski University, 25-317 Kielce, Poland; dorota1010@tlen.pl

**Keywords:** COVID-19, SARS-CoV-2, diagnostics, therapy, prevention

## Abstract

The first Polish recommendations for the management of COVID-19 were published by the Polish Society of Epidemiologists and Infectiologists (PTEiLChZ) on 31 March 2020, and the last three years ago. The emergence of new SARS-CoV-2 variants, a different course of the disease, as well as new knowledge about therapies and vaccines, requires updating diagnostic, therapeutic, and prophylactic guidelines. Despite the reduction in the threat associated with COVID-19, there is a risk of another epidemic caused by coronaviruses, which was an additional reason for developing a new version of the guidelines. In preparing these recommendations, the Delphi method was used, reaching a consensus after three survey cycles. Compared to the 2022 version, the names of the individual stages of the disease have been changed, adapting them to the realities of clinical practice, and attention was paid to the differences observed in immunosuppressed patients and in children. Some previously recommended drugs have been discontinued, including monoclonal antibodies. In addition, general principles of vaccination were presented, as well as issues related to the post-COVID syndrome.

## 1. Introduction and Rationale for Developing the Guidelines

The coronavirus disease 2019 (COVID-19) pandemic caused by severe acute respiratory syndrome coronavirus 2 (SARS-CoV-2) was declared by the World Health Organization (WHO) on 11 March 2020, and the public health emergency was terminated on 5 May 2023. However, SARS-CoV-2 infections continue to occur worldwide, although both the virus and the disease it causes have changed significantly. Despite the reduced threat associated with COVID-19, there is a high probability that sooner or later, we will face another epidemic caused by coronaviruses, as was the case with SARS in 2002 or MERS in 2012. The conviction that this event is inevitable has become one of the reasons for developing the current version of the recommendations for the management of COVID-19, which may be useful in the initial period of a possible next epidemic caused by coronaviruses. In developing these recommendations, the Delphi method was used, reaching a consensus of views after three survey cycles.

Over the past 5 years, SARS-CoV-2 infection has been confirmed in almost 800 million people worldwide, of whom over 7 million have died [1]. In Poland, nearly 7 million people have been recorded with confirmed SARS-CoV-2 infection, of whom 121 thousand have died [1]. The number of deaths due to COVID-19 has decreased significantly in the past 3 years, resulting from the evolution of the virus, which has become much less pathogenic. Currently, the percentage of pneumonia and embolic complications is very low, and respiratory failure requiring intubation is rare.

Since the creation of the first Polish recommendations for the management of patients with COVID-19 published by the Polish Society of Epidemiologists and Infectious Disease Physicians (PTEiLChZ) on 31 March 2020 [2], they have been updated twice in 2021 and 2022, and between these updates they have been annexed three times [3,4,5,6,7]. The current update of the PTEiLChZ recommendations is primarily due to a different clinical picture of the disease, resulting from the genetic variability of SARS-CoV-2, discussed in the chapter on the characteristics of the virus. In the part devoted to the clinical picture of the disease, the names of the individual phases of the disease have been changed, adapting them to the realities of clinical practice. In the section on infection diagnostics, attention was drawn to the differences observed among immunosuppressed patients. The chapter on treatment includes recommended antiviral and immunomodulatory drugs with a description of the principles of their use, resulting from current practical knowledge and characteristics of medicinal products. However, the use of monoclonal antibodies has been limited due to their significantly reduced effectiveness in therapy and pre-exposure prophylaxis. Some previously recommended drugs (e.g., anakinra) have also been abandoned due to research results indicating their ineffectiveness. Despite the formal end of the COVID-19 pandemic, we still observe new waves of illness and hospitalizations every few months. This situation has not been changed despite widely available, free, and updated vaccinations against SARS-CoV-2. These vaccinations are currently recommended for all age groups, and the principles of their conduct have also been included in the presented recommendations. Separate chapters are devoted to the differences in the clinical picture of COVID-19 in children and the consequences of COVID-19, even though they are much less frequently observed in the era of the Omicron variant dominance. At the same time, the scope of information related to intensive care has been limited to a minimum, assuming that it is associated with highly specialized, often technical procedures described in the relevant recommendations.

One should note that further updates of recommendations may be required if newer and clinically relevant (sub)lineages emerge or novel therapeutic or prophylactic options become available. It should also be emphasized that some of the recommendations presented may not be fully consistent with practices in other health care systems or their implementation may be hampered by limited resource availability.

## 2. Characterization of SARS-CoV-2: Etiology, Molecular Evolution and Pathogenesis

SARS-CoV-2, a member of the Coronaviridae family (subgenus *Sarbecovirus*), shares ~96% genetic identity with the bat virus RaTG13 [8]. Transmission to humans likely involved an intermediate host, as suggested by phylogenetic analyses, and its origin has been attributed to natural evolutionary processes [9,10,11]. The hypothesis of a laboratory leak remains unconfirmed. However, it may never be possible to fully reconstruct the events that led to the interspecies leap and virus adaptation to humans [12].

Since the beginning of the spread of SARS-CoV-2 in the human population, the virus has been subject to genetic variability and selection phenomena under the influence of various factors, such as the host organism’s environment and the impact of external factors. The evolution of SARS-CoV-2 occurs mainly through point mutations, as well as genetic recombination, and may affect the epidemiology, clinical course of infections, and the effectiveness of vaccinations and therapies. The key (although not the only) area of variability is the gene encoding the spike protein (S) responsible for binding to the cell surface receptor. Monitoring the variability of the virus allows for the separation of its phylogenetic development lines and the identification of individual subvariants. The most important of them are classified by the WHO, depending on phenotypic features and the identified epidemiological risk, into three groups: (1) Variants under Monitoring (VUM), (2) Variants of Interest (VOI), and (3) Variants of Concern (VOC). In the past, the VOC group, the most important from the public health perspective, included the Alpha (B.1.1.7), Beta (B.1.351), Delta (B.1.617.2), Gamma (P1), and Omicron (B.1.1.529) variants. However, the spread of Omicron since November 2021 has led to the displacement of all other SARS-CoV-2 lineages. As a result, in 2023, the WHO classification was updated to include only particular Omicron sublineages in the individual groups, VUM, VOC, and VOC. This approach allows for a better understanding of the impact of the ongoing variability of Omicron on its epidemiological and clinical significance and modification, depending on the needs, as well as preventive and therapeutic measures [13].

Regardless of the variant, SARS-CoV-2 released during coughing or sneezing in bioaerosol spreads mainly by airborne droplets, less frequently by dust droplets. Cells are infected by the combination of the S protein of the virus with a functional cellular receptor, angiotensin II-converting enzyme (ACE-2). Then, depending on the site of proteolytic activation of the S protein, the virus enters the cells directly through the fusion of the cell and viral membranes or by endocytosis. Strong expression of the ACE-2 receptor has been observed in the epithelial cells of the upper respiratory tract, alveolar epithelium type 1 and 2, and lung endothelium, but also in the mucous glands of the tubulovesicular glands of the upper esophagus, in enterocytes of the ileum and colon, in the kidneys, heart, pancreas, and, which seems to be a key element of pathogenesis, in the endothelium of arterial and venous vessels and smooth muscle cells of arterial vessels [14]. The multiple localizations of ACE-2 contribute to the complex and systemic clinical picture of the infection. Alternative receptors for SARS-CoV-2 have also been detected, including ASGR1, KREMEN1, histamine receptor 1, and neuropilin 1 [15,16,17].

After the virus enters, it replicates and damages cells, which can cause local inflammation and organ dysfunction. The variety and severity of symptoms result from the level of virus replication in individual patients and differences in the efficiency of the immune system. In some patients (e.g., elderly people with multiple diseases, young people with primary/secondary immune deficiency), excessive immune response associated with increased inflammation, including cytokine storm, leads to multi-organ damage, acute respiratory distress syndrome, and endothelial dysfunction, which promotes the formation of clots and other vascular complications [18,19].

The emergence and spread of the Omicron lineage brought significant changes in the pathogenesis of SARS-CoV-2, resulting from the accumulation of various mutations in its genome, including a record number of mutations in the gene encoding the S protein. Omicron, in contrast to the preceding lineages of the virus, seems to prefer the endosomal route of entry into cells, contributing less to the formation of fusional cell structures, more often infecting the upper respiratory tract, with a lower tropism to lung tissues [20,21,22]. This explains its potentially milder clinical course compared to earlier variants, such as Delta. However, the increased transmissibility of Omicron and the ability of immune escape contribute to the rapid spread of its subvariants, creating challenges in controlling the spread of infections, increasing the risk of reinfection, and necessitating an update of the antigenic composition of vaccines.

## 3. Clinical Picture of the Disease

The clinical picture of COVID-19 has changed since the first cases appeared in humans. Currently, in the period of dominance of Omicron subvariants characterized by a milder course, availability of vaccines and drugs, and a high level of immunity of the population resulting from previous infections and/or vaccinations, the overall risk of severe disease is much lower. However, SARS-CoV-2 infection in various groups of patients, including those over 60 years of age, especially with comorbidities, and pregnant women, is still the cause of hospitalization and death [23,24]. The incubation period of the disease is 3–4 days and is usually longer than in other viral respiratory infections, from which it requires differentiation [25]. Transmission after 6–7 days from the onset of symptoms is unlikely, although possible from people with immunodeficiencies, in whom viremia may persist longer [26]. The percentage of asymptomatic infections in the general population is estimated at about 32%; it is higher in vaccinated people and those under 20 years of age [27]. A characteristic feature of COVID-19 is the variety of symptoms, mostly non-specific, and their variable severity, based on which four stages of the disease can be distinguished.

Stage 1 (mild, without pneumonia) includes asymptomatic patients or those with mild symptoms and oxygen saturation (SpO_2_) ≥95% in ambient air. The most common symptoms are fever, cough, fatigue, difficulty breathing, headache, sore throat, muscle pain, nasal congestion, runny nose, nausea, vomiting, and diarrhea [23,28,29]. The disease primarily affects the upper respiratory tract, and no signs of pneumonia are found on physical examination and imaging. Anosmia or ageusia are rarely reported [23,30]. In pregnant women, fever, shortness of breath, cough, and muscle pain occur less frequently than in non-pregnant patients [24]. In older people, the absence of fever and geriatric syndromes are the most common atypical symptoms of the disease [31]. In most SARS-CoV-2 infections, the disease ends at this stage.

In stage 2 (moderate, with possible pneumonia) of disease progression, patients have clinical and imaging signs of interstitial pneumonia with SpO_2_ below 95% on ambient air. Patients in this stage may require oxygen therapy, and for this reason, especially in the presence of risk factors, hospitalization is justified. Proper management allows, in most cases, to avoid further progression of the disease.

Stage 3 (severe pneumonia with multi-organ damage and cytokine storm) is severe pneumonia accompanied by at least one of the following symptoms: respiratory rate above 30/min, severe respiratory failure, and SpO_2_ <90% in ambient air. At this stage, psychiatric and neurological complications often occur, covering a wide spectrum from faints to strokes [32,33]. There is also heart damage manifested by inflammation, ST-segment elevation myocardial infarction, arrhythmias, and pericarditis [34,35]. A biomarker of a severe course of the disease is elevated troponin concentration in blood serum [36]. It has been shown that acute myocardial damage in patients who survive does not progress, and the medium-term prognosis is favorable [37]. Almost all patients at this stage have coagulation disorders indicating intravascular coagulation and thromboembolic risk, which at least partially explain multi-organ damage.

Stage 4 (critical, with acute respiratory distress syndrome (ARDS) and multi-organ failure) develops especially in individuals with risk factors (age >60 years, obesity, diabetes, neoplastic diseases, chronic heart failure, chronic respiratory failure, chronic renal failure, immunodeficiencies, and immunosuppression). At this stage, we observe acute respiratory failure accompanied by septic shock and/or multi-organ dysfunction, including acute kidney and liver damage [28,29]. There is a high risk of arterial and venous thromboembolism, which persists for at least a year after infection [38]. Co-occurring bacterial infections are common in patients hospitalized in the ICU, and, in addition to ARDS, pneumothorax and multi-organ failure are the main causes of death [39].

## 4. Laboratory Diagnostics

The standard for confirming SARS-CoV-2 infection remains the detection of viral antigens or viral genetic material in samples taken from the nasopharynx. Importantly, in the era of hybrid immunity in the population (previous illness, vaccinations, or their combination) and circulating Omicron subvariants, the sensitivity of antigen or genetic tests changes in the following days from the onset of disease symptoms, being the highest between 3 and 4 days. For example, for antigen tests—30.0–60.0% on the first day, 59.2–74.8% on the third day, and 80.0–93.3% on the fourth day of symptoms with a sharp reduction after the 5th day of symptoms) [40].

### 4.1. Antigen Tests

In the era of circulating Omicron subvariants and hybrid immunity, antigen tests remain highly sensitive, allowing detection of most SARS-CoV-2 infections, which translates into relatively cheap diagnostics and facilitates the reduction in the risk of transmission. Testing sensitivity is lower in asymptomatic individuals [41]. Currently, antigen tests should meet the following requirements [42]:

- CE certified;
- Clinical efficacy has been confirmed on samples taken from the nasal cavity, oropharyngeal cavity, or nasopharyngeal cavity;
- Sensitivity >80% in studies on symptomatic patients in the first seven days of symptom onset or in asymptomatic individuals whose SARS-CoV-2 infection was confirmed by molecular testing;

or

- Sensitivity >90% for individuals with SARS-CoV-2 genetic material detected with a detection threshold (cycle threshold, CT) <25, reflecting high viral load.

Multiplex antigen tests are also currently in use, allowing for simultaneous detection of SARS-CoV-2 antigens, influenza (often with differentiation between influenza A and B), RSV, and other viruses associated with upper respiratory tract infections. The sensitivity of these tests is higher in people with high viral loads, but they allow for rapid differential diagnosis [43].

### 4.2. Molecular Tests

Molecular tests detecting the genetic material of the virus (NAAT—nucleic acid amplification testing) remain highly sensitive and specific. The testing has a higher sensitivity compared to antigen testing for detecting infection in the first days of illness or in asymptomatic individuals. It should be noted that the CT value can be a valuable indicator of the viral load [44]. CT values <25 reflect high SARS-CoV-2 loads, while values >35 indicate the residual presence of genetic material and are often not associated with active infection. Importantly, SARS-CoV-2 genetic material can be detectable for a long time for more than 3 months, and in immunocompromised individuals, even up to 9 months, and this is not associated with viral replication [45].

### 4.3. Serological Tests

In the era of hybrid immunity, when most of the population has been exposed to SARS-CoV-2 or vaccinated, serological tests have no practical clinical use. Serological antibody titers decline 6–9 months following SARS-CoV-2 infection or immunization, with greater decreases in vulnerable populations, especially older individuals and immunosuppressed/immunocompromised people [46]. Also, standard serological tests do not demonstrate specific immunity against new emerging SARS-CoV-2 variants. Clinically, anti-SARS-CoV-2 may be analyzed in people with immune disorders who do not produce antibodies to assess the response after vaccination or after the disease.

## 5. Treatment

**Figure 1:**
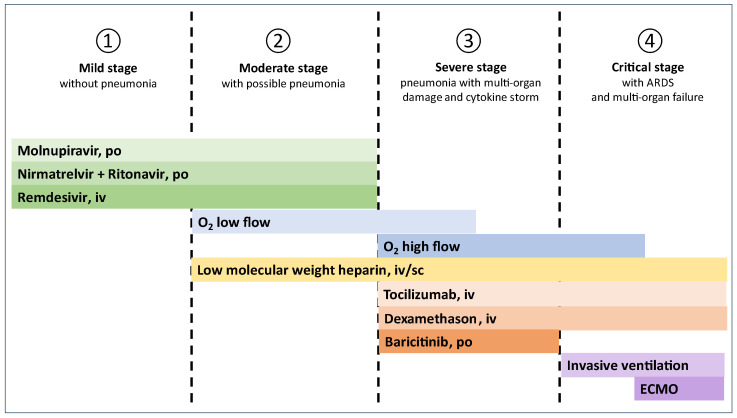
Recommended treatment regimen for COVID-19.

Therapeutic management of COVID-19 depends on the clinical stage of the disease, the patient’s condition, and the risk of severe disease. The recommended therapeutic management is presented in Figure 1, and the details are provided in Table 1. The course of the disease can be divided into four stages, which differentiate the therapeutic management (Figure 1).

### 5.1. Stage 1 (Mild, Without Pneumonia)

The vast majority of patients are infected with SARS-CoV-2 asymptomatically or with minor symptoms. The percentage of such patients varies depending on the characteristics of the dominant variant of the virus. However, a mild course of the disease may precede progression to a more severe course of the disease, especially in people belonging to risk groups. Patients in stage 1 do not require hospitalization but should remain under the care of a primary care physician, who will assess their general condition, SpO_2_, and risk factors for progression to a severe form of COVID-19, which include age >60 years, obesity, diabetes, cancer, chronic heart failure, chronic respiratory failure, chronic renal failure, immunodeficiencies, and immunosuppression. Patients belonging to any of these groups, regardless of their clinical condition, should receive antiviral therapy with molnupiravir, nirmatrelvir/ritonavir, or remdesivir (alphabetical order, does not indicate priority of selection) as soon as possible to reduce the risk of disease progression and the need for hospitalization. Treatment should be administered within 5 days of the onset of symptoms [47,48,49,50,51,52,53,54,55,56,57]. In patients with immunosuppression resulting from a concomitant disease or therapy, the time of initiation of antiviral treatment may be extended to 10 days from the onset of COVID-19 symptoms due to the longer period of virus replication. During antiviral therapy [58,59,60] and immediately after its completion, effective contraception is recommended for women of childbearing age, although the use of remdesivir is permissible in the 2nd and 3rd trimester of pregnancy and for breastfeeding women. When planning the use of nirmatrelvir/ritonavir, the risk of interactions with other drugs taken by the patient should be assessed using online tools [61]. Details of the dosage and contraindications to the use of antiviral medications are presented in Table 1. In patients without risk factors, with mild symptoms of respiratory tract infection, such as fever, sore throat, headache, muscle pain, and rhinitis, antiviral treatment may be considered based on the patient’s clinical condition, epidemiological situation, and drug availability. As part of symptomatic treatment, patients may require the use of antipyretics (paracetamol) and antitussives. Inhaled budesonide can be used as a supportive treatment to alleviate the course of the disease [62,63]. In patients with stage 1, systemic glucocorticosteroids are contraindicated due to their immunosuppressive effects, which may intensify and prolong viral replication and thus worsen the prognosis [64]. In chronically immobilized patients and with other indications for antithrombotic prophylaxis unrelated to COVID-19, and especially in patients with risk factors for deep vein thrombosis and/or pulmonary embolism, the use of low-molecular-weight heparin in prophylactic doses is indicated [65,66]. There is no evidence of the benefits of antibiotics in this stage of the disease. Therefore, antibiotic therapy should be considered only in the case of diagnosed or suspected bacterial infection of the respiratory tract resulting from the clinical picture, microbiological, and/or imaging tests [67,68,69].

### 5.2. Stage 2 (Moderate, with Possible Pneumonia)

Clinical deterioration, especially with the occurrence of dyspnea requiring oxygen therapy and a decrease in SpO_2_ below 95%, is an indication for hospitalization. Patients with comorbidities, in whom the course of the underlying disease has worsened during the SARS-CoV-2 infection, should be referred to the hospital to the department appropriate for the treatment of this disease, even in the absence of indications for oxygen therapy. In such cases, antiviral therapy, if initiated as part of primary health care, should be continued. Usually, in this phase of the disease, low-flow oxygen therapy, not exceeding 15 L/min, is sufficient. Prophylactic doses of low-molecular-weight heparin are part of the standard treatment for hospitalized patients, with the option of increasing to therapeutic doses in justified cases. If the 5th day has not passed since the onset of COVID-19 symptoms (10th day in immunosuppression), antiviral treatment should be initiated [58,59,60]. In the case of hospitalized patients, oral nirmatrelvir/ritonavir or molnupiravir or intravenous remdesivir should be used (alphabetical order, does not indicate priority of selection) [49,50,51,52,58,59,60]. Antiviral therapy in hospital settings is also recommended for patients who do not require oxygen therapy to prevent progression associated with risk factors or to limit the spread of infection among other patients and staff. Remdesivir can be administered regardless of oxygen therapy and nirmatrelvir/ritonavir before use. Adding glucocorticosteroids to antiviral drugs at this stage of the disease is not beneficial, and due to its immunosuppressive effect, it may intensify or prolong viral replication. However, in the absence of clinical improvement despite antiviral therapy, dexamethasone may be considered in the second week of the disease [64]. In the case of a diagnosed or suspected bacterial infection resulting from the clinical picture, microbiological tests, and/or imaging, the use of antibiotics may be considered [69]. Limitations in the use of drugs and details of dosage are presented in the discussion of stage 1 and in Table 1.

### 5.3. Stage 3 (Severe, Pneumonia with Multi-Organ Damage and Cytokine Storm)

Clinical deterioration may occur at the beginning of the 2nd week of the disease with increasing dyspnea and SpO_2_ reduction below 90%, which in some patients requires high-flow oxygen therapy up to 60 L/min. At the same time, this may indicate the beginning of a cytokine storm, which will be confirmed by an increase in the concentration of interleukin 6 (IL-6) in the blood. Exceeding the concentration of 100 pg/mL justifies the administration of tocilizumab, a monoclonal antibody directed against the IL-6 receptor, which significantly reduces the risk of mechanical ventilation and death [70,71,72,73,74,75,76]. Tocilizumab should be administered by intravenous infusion at a dose depending on body weight. If there is no effect, another infusion can be administered after 8–24 h, but the benefits of administering two doses in comparison with a single dose have not been proven [76]. The use of intravenous glucocorticosteroids in a daily dose not exceeding 6 mg of dexamethasone is indicated when the clinical condition deteriorates without an increase in IL-6 concentration or when the use of tocilizumab has not been effective [72,77]. The use of higher doses of dexamethasone worsens the prognosis [78,79]. An alternative to tocilizumab may be the orally administered Janus kinase inhibitor baricitinib, which has been proven effective, especially in patients requiring high-flow oxygen therapy [80,81,82,83]. Details of the dosage and contraindications of the drugs recommended in stage 3 are presented in Table 1. Combination therapy with the abovementioned drugs is not recommended due to the lack of data from clinical trials. At this stage of the disease, patients should continue to receive low-molecular-weight heparin. The use of antiviral drugs at this stage of the disease is not justified, except for immunosuppressed patients who are at risk of prolonged persistence of SARS-CoV-2 viremia. Antibiotic therapy is justified only in the case of a high probability of bacterial infection resulting from the clinical picture, microbiological, and/or imaging tests [69].

**Table 1:** Recommended therapeutic procedures, including basic and supportive treatment in individual stages of COVID-19.

***Stage 1 (mild, without pneumonia)***	
1st week of the disease SpO_2_ ≥95% primary health care (general practitioners)	
**Basic treatment**	**Supportive treatment**
**Antiviral drugs** (alphabetical order, does not indicate priority of selection). Initiation of antiviral therapy within 5 days of symptom onset and within 10 days in immunosuppressed patients. Recommended for patients at risk of severe COVID-19 * and to be considered in other patients. **Molnupiravir**, adults only, oral, 2 times daily, 800 mg for 5 days. Contraindicated in pregnant and lactating women [58]. OR **Nirmatrelvir/ritonavir**, adults only, orally, twice daily, 300/100 mg for 5 days. Drug interactions should be checked [61]. Contraindications: - in pregnant and lactating women - in Child–Pugh C liver failure, - contraindicated at eGFR <30 mL/min, and at eGFR 30–60 mL/min, dose reduction to 150/100 mg [59]. OR **Remdesivir**, adults and children with body weight >40 kg, intravenously, once daily for 3 days, on day 1, 200 mg, on day 2 and 3, 100 mg. Contraindicated in women in the first trimester of pregnancy; permissible in the 2nd and 3rd trimesters and in breastfeeding women [60].	rest, oral hydration, inhaled budesonide, 2 × 800 μg daily [62], antipyretics (paracetamol, ibuprofen, etc.), antitussives in case of persistent cough, low-molecular-weight heparin in chronically immobilized patients and with other indications for antithrombotic prophylaxis unrelated to COVID-19.
**Notes:**	
Systemic glucocorticosteroids are contraindicated. Antibiotic ed therapeutic procedures, including basic and supportive treatbacterial respiratory infection.	
***Stage 2 (moderate, with possible pneumonia)***	
1st–2nd week of the disease SpO_2_ <95% hospitalization	
**Basic treatment**	**Supportive treatment**
**Oxygen therapy:** low-flow, up to 15 L/min. **Antithrombotic drugs:** Low-molecular-weight heparin in a prophylactic dose, which can be increased in justified cases. **Antiviral drugs** (alphabetical order, does not indicate priority of selection). Initiation of antiviral therapy within 5 days of the onset of symptoms and within 10 days in immunosuppressed patients. **Molnupiravir**, adults only, orally, twice daily, 800 mg for 5 days. Contraindicated in pregnant and lactating women [58]. OR **Nirmatrelvir/ritonavir**, adults only, orally, twice daily, 300/100 mg for 5 days. Drug interactions should be checked [61]. Contraindications: - in pregnant and lactating women - in Child–Pugh C liver failure, - contraindicated at eGFR <30 mL/min, and at eGFR 30–60 mL/min, dose reduction to 150/100 mg, [59] OR **Remdesivir**: - adults and children with body weight >40 kg, intravenously, once a day for 5–10 days, 200 mg on the 1st day and 100 mg on subsequent days. - children >4 weeks with bodyweight 3–40 kg, intravenously, once a day for up to 10 days, on the 1st day 5 mg/kg, on subsequent days 2.5 mg/kg. Contraindicated in women in the 1st trimester of pregnancy; permissible in the 2nd and 3rd trimesters and for breastfeeding women [60].	symptomatic treatment oral or intravenous hydration
**Notes:**	
Glucocorticosteroids can be considered in the 2nd week of illness, but only if there is no clinical improvement despite the use of antiviral drugs and oxygen therapy; oral or intravenous dexamethasone in adults, 4–8 mg/day, in children, 0.1–0.15 mg/kg (maximum 6 mg) daily, for no longer than 10 days. Antibiotic therapy only if there is a diagnosis or reasonable suspicion of an overlapping bacterial infection of the respiratory tract.	
***Stage 3 (severe, pneumonia with multi-organ damage and cytokine storm)***	
2nd week of the disease SpO_2_ <90% hospitalization	
**Basic treatment**	**Supportive treatment**
**Oxygen therapy:** high-flow, up to 60 L/min. **Antithrombotic drugs:** Low-molecular-weight heparin in a prophylactic dose, which can be increased in justified cases. **Glucocorticosteroids:** Dexamethasone administered intravenously in adults, 6 mg, in children, 0.1–0.15 mg/kg (maximum 6 mg) daily, for no longer than 10 days [78,79]. **Immunomodulatory drugs:** **Tocilizumab**, adults with IL-6 levels >100 pg/mL, 60 min intravenous infusion, 8 mg/kg, and if no improvement, the 2nd dose may be repeated after 8–24 h. Contraindications: - neutrophil count <1000/μL - platelets <50 thousand/μL, - alanine aminotransferase activity >10 times the upper limit of normal [76]. OR **Baricitinib**, adults, oral, once daily, 4 mg, up to 14 days. Contraindications: - neutropenia <500/μL, - lymphopenia <200/μL, - eGFR <15 mL/min and dialysis patients [80]	symptomatic treatment intravenous hydration
**Notes:**	
Antibiotic therapy only if there is a diagnosis or reasonable suspicion of an overlapping bacterial infection of the respiratory tract.	
***Stage 4 (critical, with ARDS and multi-organ failure)***	
2nd–3rd week of the disease need for mechanical ventilation intensive care unit	
**Basic treatment**	**Supportive treatment**
**Oxygen therapy:** High-flow oxygen therapy: up to 60 L/min OR Invasive ventilation OR Extracorporeal venovenous transmembrane oxygenation (VV ECMO) in selected patients, **Antithrombotic drugs:** Low molecular weight heparin in a prophylactic dose, which can be increased in justified cases. **Glucocorticosteroids:** Dexamethasone, adults, intravenous, daily dose of 12 mg, in children, 0.1–0.15 mg/kg (maximum 6 mg) daily, for no longer than 10 days. If dexamethasone is not available, other glucocorticoids in equivalent doses may be given. AND/OR **Immunomodulatory drugs:** **Tocilizumab**, adults, if not previously administered, then on the first day of mechanical ventilation, a 60 min intravenous infusion of 8 mg/kg. Contraindicated in patients with: - neutrophil count <1000/μL, - platelets <50 thousand/μL, - alanine aminotransferase activity >10 times the upper limit of normal [76]. OR **Baricitinib**, adults, oral (intragastric), once daily, 4 mg, up to 14 days. Contraindicattions: - neutropenia <500/μL, - ymphopenia <200/μL, - eGFR <15 mL/min and dialysis patients [80].	symptomatic treatment intravenous hydration
**Notes:**	
Antibiotic therapy only if there is a diagnosis or reasonable suspicion of an overlapping bacterial infection of the respiratory tract.	

* Age >60 years, obesity, diabetes, cancer, chronic heart failure, chronic respiratory failure, chronic renal failure, immunodeficiencies, immunosuppression.

### 5.4. Stage 4 (Critical, with Acute Respiratory Distress Syndrome (ARDS) and Multi-Organ Failure)

The deterioration of the patient’s condition despite the treatment and high-flow oxygen therapy usually means ARDS. A patient at this stage requires invasive ventilation of the lungs, as well as the use of glucocorticosteroids [64,84]. If the patient has not previously received tocilizumab or baricitinib, one of these immunomodulators may be considered [76,80]. There is no justification for starting or continuing antiviral treatment in patients invasively ventilated, except for patients immunosuppressed due to underlying disease or treatment. Invasive mechanical ventilation should be used in intensive care units, by established standards of care [85,86]. The use of veno-venous extracorporeal membrane oxygenation (VV ECMO) may be considered only in selected patients and should be performed in accordance with international guidelines in centers with appropriate experience and technical capabilities [86,87].

## 6. Therapies with Unproven Effectiveness

In the initial period of the pandemic, the use of numerous drugs in COVID-19 therapy was considered, which were primarily supposed to demonstrate antiviral activity. In the case of some therapies, the effectiveness was assessed in clinical trials, although only for individual ones, and these were randomized controlled trials. Of course, the negligible strength of evidence for observational studies or case series does not allow for including such therapies in recommendations. For the drugs presented below, their effectiveness has not been confirmed so far; their ineffectiveness has been shown, or their use has been shown to be not safe.

### 6.1. Antiviral/Anti-Infective Drugs

■ Favipiravir (the major supporting article was retracted).
■ Oseltamivir and zanamivir [88].
■ Amantadine and rimantadine [89].
■ Antiretroviral drugs (used to treat HIV infection) [90].
■ Ivermectin [91].
■ Fluvoxamine [92].
■ Interferons.
■ Intravenous immunoglobulin and specific immunoglobulin against SARS-CoV-2 [93].
■ Anti-SARS-CoV-2 monoclonal antibodies—bamlaniwimab/etesewimab, casirivimab/imdewimab, thixagevimab/cilgavimab—lack of effectiveness against the Omicron variant of SARS-CoV-2 [94];
■ Convalescent plasma [95].

### 6.2. Anti-Inflammatory Drugs

■ Nonsteroidal anti-inflammatory drugs (NSAIDs) can be used for symptomatic treatment in therapeutic doses and for short periods [96];
■ Anakinra, although initially recommended for use in stage 3, ultimately failed to demonstrate efficacy in clinical trials [97];
■ Glucocorticosteroids are contraindicated in stage 1 of the disease.

### 6.3. Other Medications

■ Metformin [98];
■ Dietary supplements, including vitamins C and D and zinc [99];
■ Azithromycin and other antibiotics should only be used in the presence of a concomitant bacterial infection [100];
■ Chloroquine and hydroxychloroquine [101].

In case of infection in patients previously taking immunosuppressive drugs or any drugs for comorbidities, it is recommended to maintain these therapies during COVID-19.

## 7. Post-COVID Syndrome

The term “post-COVID syndrome” refers to persistent symptoms or organ dysfunction following the acute phase of COVID-19 [102]. In October 2021, the WHO published a case definition for post-COVID syndrome, which indicates that it can be diagnosed in patients with probable or confirmed SARS-CoV-2 infection. Its symptoms usually appear after 3 months of COVID-19 onset, last at least 2 months, and are not associated with another diagnosis [103]. In the new ICD-10-CM classification, the post-COVID syndrome has been assigned the code U09.9.

The incidence of the syndrome ranges from 10% to 60% and depends on many factors, such as gender, the presence of chronic diseases or immune deficiencies, the severity of the infection, the antiviral treatment used in the acute phase, and anti-SARS-CoV-2 vaccination. Meta-analyses show that the most common symptoms include fatigue (32–57%), sleep disorders (10–55%), shortness of breath (17–38%), weakness (8–56%), chest pain (11–24%), headaches (9–27%), and joint pain (7–24%). [104]. Pediatric multisystem inflammatory syndrome (PIMS/MIS-C) is a distinct condition that appears after COVID-19 in children and young adults who meet specific diagnostic criteria [105]. The results of the Polish prospective study SILCOV-19 (The Silesian Complications of COVID-19 Database) confirm that the most common symptoms of post-COVID-19 syndrome are fatigue, shortness of breath, palpitations, and smell and taste disorders. [106].

Cardiac manifestations occur in about 15% of post-COVID-19 patients [107]. These include mainly chest pain (OR 4.0), palpitations (OR 3.4), and hypertension (OR 1.7). One of the defined post-COVID-19 phenotypes is chronic fatigue syndrome (ME/CFS). Post-COVID syndrome is more common in women [108] and in patients with immune disorders and chronic diseases, including bronchial asthma, chronic lung disease, type 2 diabetes, heart failure, and chronic kidney disease [109]. Post-COVID syndrome symptoms usually regress over time, although it has been shown that most patients with this diagnosis continue to report symptoms in the second year after diagnosis [110].

There is no evidence to recommend routine screening in asymptomatic individuals. Diagnostics should be directed at the symptoms reported [111]. Patient education is key, particularly regarding the symptoms of the post-COVID syndrome. Objective methods of assessing lung disease include the Borg dyspnea scale, home pulse oximetry, the 6 min walk test (6MWT), and lung function tests. In the case of cardiac symptoms, 24 h Holter monitoring and echocardiography are used. There are currently no recommendations for routine assessment of coagulation parameters after COVID-19.

Rest, physical activity, and pulmonary rehabilitation play an important role in recovery from COVID-19. No evidence supports routine thromboprophylaxis, although high-risk patients may require anticoagulation for up to 30 days after discharge, according to general recommendations [112,113]. Randomized studies have shown a beneficial effect of pulmonary rehabilitation combined with aerobic activity [114] and controlled weight loss in overweight individuals [115] on reducing post-COVID symptoms.

Post-COVID syndrome develops less frequently in people vaccinated against COVID-19 both before and after the disease [116], as well as in those who received targeted antiviral treatment in the acute phase of infection [117]. Currently, these activities are a key element of the strategy to combat post-coital syndrome. Randomized studies have not confirmed the clinical efficacy of some drugs postulated as effective in the treatment of post-COVID syndrome (including losartan and imatinib); further studies are ongoing.

## 8. Clinical and Therapeutic Differences in Children

Initially, low rates of infection in children increased significantly during the period of dominance of the Omicron variant. According to data from the UK, in November–December 2021, seropositivity rates were 37% in children aged 1–4 years, 54% in children aged 5–11 years, 78% in children aged 12–15 years, and 87% in children aged 16–17 years. By September 2022, seroprevalence had increased to 93%, 98%, 99%, and 99%, respectively [118]. In a Polish study assessing the seroprevalence of IgG antibodies against SARS-CoV-2 in 686 children hospitalized for reasons other than COVID-19 from 1 June 2021, to 30 April 2022, the presence of these antibodies was shown in 57% of hospitalized patients and an increase in the percentage to 87.5% during the fourth and fifth waves of COVID-19 (Delta and Omicron variants). The vast majority of parents had no knowledge of COVID-19 infection in their children, which may indicate an asymptomatic infection or a mild course of the disease [119].

Children usually show milder symptoms or remain asymptomatic. Pathophysiological mechanisms responsible for the mild course of the disease in children include lower expression of the ACE2 receptor, lower binding affinity between ACE2 receptors and spike proteins, strong innate and adaptive immune responses, physiologically high percentages of lymphocytes in younger children, fewer comorbidities, and significant cross-immunity due to previous exposure to other coronaviruses. Higher levels of IgM, IgG, and interferon and lower IL-6 and IL-10 were observed in children with COVID-19 [120]. The clinical presentation of COVID-19 was associated with age and the dominant SARS-CoV-2 variant at the time of infection. The risk of hospitalization in childhood due to severe COVID-19 has always been very low, and the length of hospital stay in most children was 1–2 days. A higher risk of hospitalization was observed in infants and children with comorbidities. The risk of hospitalization was lower with the Omicron variant compared to earlier SARS-CoV-2 variants [121].

Analysis of the course of COVID-19 in 1283 Polish children, covering the period from March 1, 2020, to December 31, 2020, showed that the most common symptom was fever (46%). In the youngest children, fever, rhinitis, and diarrhea were more frequently observed, while adolescents more often complained of headache, sore throat, impaired sense of smell and taste, and weakness. One-fifth of patients remained asymptomatic. Pneumonia was diagnosed in 12% of patients, more often in younger children. During the second wave, infected patients were younger and required longer hospitalization [122]. COVID-19 in infants manifested itself as a mild infection of the gastrointestinal tract or respiratory system, but pneumonia with decreased saturation requiring oxygen therapy was also observed [123].

The risk of PIMS (pediatric inflammatory multisystem syndrome) after SARS-CoV-2 infection decreased from 0.038% during the Alpha variant dominance to 0.026% during the Delta variant dominance. Currently, the risk of PIMS is <0.01%, which may be due to genetic changes in the SARS-CoV-2 surface antigens responsible for the hyperinflammatory immune response [124]. Considering that almost all children have antibodies against SARS-CoV-2, mainly following infection but also vaccination, a small percentage of more severe disease courses, including myocarditis and PIMS, is expected [125].

Most pediatric patients with COVID-19 require only symptomatic treatment. Antiviral drugs (remdesivir) and glucocorticosteroids are reserved for severe cases, especially in patients requiring oxygen therapy [126]. Significant risk factors for post-COVID syndrome in children were age >12 years, comorbidities, and female gender. Vaccination was associated with a lower risk of post-COVID syndrome in older children and a lower risk of reinfection [127]. The COVID-19 pandemic has significantly affected the mental health of children due to restrictions resulting from social distancing and distance learning. An increase in anxiety disorders and depression is observed in children and adolescents [128]. It is believed that the increase in the incidence of obesity in the pediatric population over the past decade has been amplified by the pandemic [129]. Undoubtedly, the immunological consequences caused by the COVID-19 pandemic are related to the observed changes in the epidemiological and clinical picture of many infectious diseases in children.

## 9. Vaccinations

Vaccinations against COVID-19 remain a key element in the prevention of severe disease, hospitalization, and deaths due to it [130,131,132]. Vaccination recommendations are adapted to the epidemiological situation, the evolution of the SARS-CoV-2 virus, and the availability of vaccines. Regular introduction of new vaccines against COVID-19 adapted to the circulating variants of the virus [130,131,132] is expected. These vaccines will take into account the updated recommendations of the WHO and the European Medicines Agency and will be developed based on mRNA or subunit technology [133]. Below is the most important information and recommendations for vaccinations in Poland, current as of the date of publication of these recommendations.

### 9.1. Available Vaccines

In the 2024/2025 season, the following COVID-19 vaccines are registered in the European Union:

- mRNA vaccines:
  - Comirnaty (Pfizer-BioNTech);
  - Spikevax (Moderna).
- Subunit protein vaccines (not available in Poland):
  - Nuvaxovid (Novavax)—containing recombinant S protein and Matrix-M adjuvant;
  - Bimervax (Hipra)—containing recombinant S protein and SQBA adjuvant.

All of the above vaccines are monovalent, directed against the dominant JN.1 subvariant of SARS-CoV-2 [130,134,135,136].

### 9.2. Target Groups for Vaccinations

Vaccinations against COVID-19 are recommended primarily for people at increased risk of severe disease, including:

- People aged ≥60 years;
- People with chronic diseases, including diabetes, lung diseases, kidney diseases, cardiovascular diseases, obesity (BMI ≥25), neurodevelopmental disorders, active neoplastic disease, or immunosuppression (resulting from disease or treatment);
- People staying in long-term care facilities;
- Pregnant women, due to the reduction in COVID-19 complications among newborns;
- People working in health care or long-term care facilities;
- Children aged 6 months to 11 years, especially those with chronic diseases [130,137,138,139].

### 9.3. Vaccination Schedule

A simplified vaccination schedule against COVID-19 is currently recommended:

- For individuals aged ≥12 years without significant risk factors: a single dose of the vaccine once a year, regardless of previous vaccination history and COVID-19 infections. The interval from the previous dose or infection should be at least 3 months [131,132,137].
- For individuals with severe immunodeficiency, it is recommended to administer the vaccine every 6 months, with a minimum interval of 2 months between doses. If possible, optimally ≥2 weeks before starting/continuing immunosuppressive therapy.
- Revaccination is recommended for patients vaccinated before or during hematopoietic cell transplantation or CAR T cell therapy ≥ 3 months after the procedure. Revaccination should be considered in patients vaccinated against COVID-19 during treatment with B cell-depleting therapies—vaccination is recommended 6 months after therapy. In the case of planned B cell-depleting therapy, COVID-19 vaccination should be administered 4 weeks before its initiation or resumption [131].
- For children aged 6 months to 11 years: for those vaccinated for the first time, we recommend 2 doses 4 weeks apart, and for children with severe immunodeficiency—3 doses (the first two 4 weeks apart, the third 2 months after the second dose). If possible, optimally ≥2 weeks before starting/continuing immunosuppressive therapy. Children with immunosuppression should receive subsequent doses of the vaccine every 6 months.
- It is safe and recommended to administer the COVID-19 vaccine concurrently with an inactivated influenza vaccine or a pneumococcal vaccine (in one visit), as well as other routinely administered vaccines.
- COVID-19 vaccines can be administered at any interval from other vaccines (including those recommended during pregnancy), except for the monkeypox vaccine (MPox), in which case a minimum interval of 4 weeks should be maintained.
- Vaccines are administered intramuscularly; we recommend continuing vaccinations with a vaccine from the same manufacturer whenever possible. It is not recommended to use vaccines with an outdated composition.

### 9.4. Contraindications and Situations Requiring Caution

Contraindications to vaccination include:

- Severe allergic reaction (e.g., anaphylaxis) after the previous dose of the vaccine or to any of its components.
- Acute febrile illness or exacerbation of a chronic disease—vaccination should be postponed until the symptoms have subsided.

Particular caution should be exercised in individuals who have experienced the following after the previous dose of the vaccine:

- Myocarditis or pericarditis.
- Multisystem inflammatory syndrome (MIS-C in children or MIS-A in adults).
- We recommend monitoring all vaccinated individuals for at least 15 min after vaccination.

### 9.5. Vaccine Safety

COVID-19 vaccines are safe and generally well tolerated. The most common adverse reactions following vaccination (ADRs) include:

- Pain, redness, or swelling at the injection site.
- Fatigue, headache, muscle or joint pain, fever.

Rarely, serious ADRs such as myocarditis or pericarditis are observed, occurring mainly in young men aged 12–39 years. However, this risk is very low, and the benefits of vaccination far outweigh the potential risk [132,140,141]. Rare thromboembolic events known as vaccine-induced immune thrombotic thrombocytopenia (VITT), which involve antibodies against platelet factor 4 (PF4), have mostly been linked to adenoviral vector vaccines. These events are rarely reported after mRNA vaccines, indicating fundamental differences in the immunological mechanisms triggered by these distinct vaccine platforms.

### 9.6. Organization of Vaccinations

Vaccinations against COVID-19 should be financed by the Ministry of Health, available free of charge to the widest possible population, and carried out in:

- Primary health care (family doctors);
- Public pharmacies;
- Hospitals.

COVID-19 vaccinations are highly effective in preventing hospitalizations and deaths due to COVID-19. Vaccines have a good safety profile and are well tolerated, and possible adverse reactions following vaccination are usually mild and transient [132,140,141].

## 10. Pre-Exposure Prophylaxis

Despite the much milder course of infection with the currently dominant SARS-CoV-2 variants, pre-exposure prophylaxis should be considered in people with moderate or severe immunodeficiency or with multimorbidity at risk of a severe course of COVID-19, especially if for some reason they have not been vaccinated. The most effective form of specific prophylaxis is vaccination, which was discussed earlier. Monoclonal antibodies became useless in pre-exposure prophylaxis with the dominance of the Omicron variant, but they may be considered in people at risk of a severe course of COVID-19 provided that scientific evidence of the effectiveness of a specific drug against the currently dominant SARS-CoV-2 variant appears [142,143]. However, due to the variability of virus variants, the usefulness of drugs based on monoclonal antibodies is expiring very quickly, and the latest example is pemivibart, which was authorized by the US Food and Drug Administration (FDA) in March 2024; however, it is not subject to further expansion [144,145]. The efficacy of pharmacological prophylaxis based on the use of nirmatrelvir/ritonavir has not been confirmed, although a reduction in the risk of relapse of infection with the Omicron variant has been demonstrated [146]. As part of non-specific passive prophylaxis, it is recommended to avoid contact with infected persons, wear FFP2 or FFP3 protective masks, avoid gatherings in closed rooms, ensure adequate ventilation of rooms, frequent hand washing, and in extreme cases, isolation until the causes and effects of immunodeficiency have subsided. It seems important to note that COVID-19 patients who have performed resistance and endurance exercises in the past are at a lower risk of hospitalization and mortality, but the mechanism of this phenomenon requires further research [147].

## Data Availability

No original research data were created or analyzed in this study. Data sharing is not applicable to this article.

## Supplementary Materials

The following supporting information can be downloaded at: https://www.mdpi.com/article/10.3390/jcm14072305/s1. (Polish language version).
