# Supplementary material for: Management of SARS-CoV-2 Infection-Clinical Practice Guidelines of the Polish Association of Epidemiologists and Infectiologists, for 2025
Source: J Clin Med. 2025 Mar 27;14(7):2305. doi: 10.3390/jcm14072305 (PMC11989246; doi:10.3390/jcm14072305)
Supplement: Supplementary file 1 [file jcm-14-02305-s001.zip › jcm-3538777-supplementary.pdf]

Journal of
Clinical Medicine

MDPI

Review

Postępowanie w zakażeniach wirusem SARS-CoV-2. Wytyczne praktyki klinicznej Polskiego Towarzystwa Epidemiologów i Lekarzy Chorób Zakaźnych na rok 2025

Robert Flisiak [1,] *, Jerzy Jaroszewicz [2], Dorota Kozielewicz [3], Ernest Kuchar [4], Miłosz Parczewski [5], Małgorzata Pawłowska [3], Anna Piekarska [6], Piotr Rzymski [7], Krzysztof Simon [8], Krzysztof Tomasiewicz [9] and Dorota Zarębska-Michaluk [10]

[1] Klinika Chorób Zakaźnych i Hepatologii, Uniwersytet Medyczny w Białymstoku, Białystok.
[2] Klinika Chorób Zakaźnych i Hepatologii, Śląski Uniwersytet Medyczny, Katowice, Polska
[3] Katedra Chorób Zakaźnych i Hepatologii, Wydział Lekarski Collegium Medicum, Uniwersytet im. Mikołaja Kopernika, Bydgoszcz,
[4] Kliniki Pediatrii z Oddziałem Obserwacyjnym, Warszawski Uniwersytet Medyczny, Warszawa,
[5] Klinika Chorób Zakaźnych, Tropikalnych i Nabytych Niedoborów Odporności, Pomorski Uniwersytet Medyczny, Szczecin,
[6] Klinika Chorób Zakaźnych i Hepatologii, Uniwersytet Medyczny w Łodzi, Łódź,
[7] Zakład Medycyny Środowiskowej, Uniwersytet Medyczny w Poznaniu
[8] Klinika Chorób Zakaźnych i Hepatologii, Uniwersytet Medyczny we Wrocławiu, Wrocław,
[9] Klinika Chorób Zakaźnych i Hepatologii, Uniwersytet Medyczny w Lublinie, Lublin,
[10] Zakład Chorób Zakaźnych i Alergologii, Uniwersytet im. Jana Kochanowskiego, Kielce.
* Korespondencja: robert.flisiak1@gmail.com (RF)

Abstract: Pierwsze polskie zalecenia postępowania w COVID-19 zostały opublikowane przez Polskie Towarzystwo Epidemiologów i Lekarzy Chorób Zakaźnych (PTEiLChZ) 31 marca 2020 r., a ostatnie trzy lata temu. Pojawianie się nowych wariantów SARS-CoV-2, inny przebieg choroby, a także nowa wiedza o terapiach i szczepionkach wymagają aktualizacji zaleceń diagnostycznych, terapeutycznych i profilaktycznych. Pomimo zmniejszenia zagrożenia związanego z COVID-19, istnieje ryzyko kolejnej epidemii powodowanej przez koronawirusy, co było dodatkowym powodem opracowania nowej wersji zaleceń. Przygotowując niniejsze rekomendacje, zastosowano metodę delficką, osiągając konsensus poglądów po trzech cyklach ankietowych. W porównaniu z wersją z 2022 roku zmieniono nazewnictwo poszczególnych stadiów choroby, dostosowując je do realiów praktyki klinicznej, zwrócono uwagę na odrębności obserwowane u chorych w immunosupresji oraz u dzieci. Zrezygnowano także z niektórych wcześniej zalecanych leków, w tym z przeciwciał monoklonalnych. Ponadto przedstawione zostały ogólne zasady szczepień, a także zagadnienia związane z zespoołem pokowidowym.

Słowa kluczowe: COVID-19, SARS-CoV-2, diagnostyka, leczenie, profilaktyka.

Academic Editor: Firstname Lastname

Received: date
Revised: date
Accepted: date
Published: date

Citation: To be added by editorial staff during production.

Copyright: © 2025 by the authors. Submitted for possible open access publication under the terms and conditions of the Creative Commons Attribution (CC BY) license (https://creativecommons.org/licenses/by/4.0/).

1. Wstęp i uzasadnienie opracowania wytycznych

Pandemia choroby koronawirusowej 2019 (COVID-19) wywołanej przez koronawirusa ciężkiego ostrego zespołu oddechowego 2 (SARS-CoV-2) została ogłoszona przez Światową Organizację Zdrowia (WHO) 11 marca 2020 r., a stan zagrożenia zdrowia publicznego został odwołany 5 maja 2023 r. Jednak zakażenia SARS-CoV-2 nadal występują na świecie, aczkolwiek zarówno wirus, jak i choroba, którą on wywołuje,

istotnie się zmieniły. Pomimo zmniejszenia zagrożenia związanego z COVID-19, istnieje wysokie prawdopodobieństwo, że wcześniej czy później czeka nas kolejna epidemia wywołana przez koronawirusy, tak jak miało to miejsce w przypadku SARS w 2002 r., czy MERS w 2012 r. Przeświadczenie o nieuchronności tego zdarzenia stało się jednym z powodów opracowania aktualnej wersji zaleceń postępowania w COVID-19, które mogą być przydatne w początkowym okresie ewentualnej kolejnej epidemii wywołanej przez koronawirusy. Opracowując niniejsze rekomendacje, zastosowano metodę delficką, osiągając konsensus poglądów po trzech cyklach ankietowych.

W ciągu ostatnich 5 lat zakażenie SARS-CoV-2 potwierdzono u prawie 800 milionów ludzi na świecie, z których ponad 7 milionów zmarło [1]. W Polsce dotychczas odnotowano blisko 7 milionów osób z potwierdzoną infekcją SARS-CoV-2, z których zmarło 121 tysięcy [1]. Liczba zgonów z powodu COVID-19 istotnie spadła w ostatnich 3 latach, co jest wynikiem ewolucji wirusa, który stał się zdecydowanie mniej patogenny. Obecnie odsetek zapaleń płuc, powikłań zatorowych jest bardzo niski a niewydolność oddechowa wymagająca intubacji należy do rzadkości.

Od czasu stworzenia pierwszych polskich rekomendacji dotyczących prowadzenia pacjentów z COVID-19 opublikowanych przez Polskie Towarzystwo Epidemiologów i Lekarzy Chorób Zakaźnych (PTEiLChZ) 31 marca 2020 r. [2] były one 2-krotnie aktualizowane w latach 2021 i 2022, a pomiędzy tymi aktualizacjami 3-krotnie aneksowane [3-7]. Obecna aktualizacja rekomendacji PTEiLChZ wynika przede wszystkim z innego obrazu klinicznego choroby, będącego wynikiem zmienności genetycznej SARS-CoV-2, omówionej w rozdziale poświęconym charakterystyce wirusa. W części poświęconej obrazowi klinicznemu choroby zmieniono nazewnictwo poszczególnych faz choroby, dostosowując je do realiów praktyki klinicznej. W części poświęconej diagnostyce zakażenia zwrócono uwagę na odrębności obserwowane wśród chorych w immunosupresji. Rozdział poświęcony leczeniu uwzględnia zalecane leki przeciwwirusowe oraz immunomodulacyjne wraz z opisem zasad ich stosowania, wynikającym z aktualnej wiedzy praktycznej i charakterystyk produktów leczniczych. Ograniczono natomiast zastosowanie przeciwciał monoklonalnych, ze względu na ich istotnie zmniejszoną skuteczność w terapii i profilaktyce przedekspozycyjnej. Zrezygnowano także z niektórych wcześniej zalecanych leków (na przykład anakinra) ze względu na wyniki badań wskazujące na ich nieskuteczność. Pomimo formalnego zakończenia pandemii COVID-19, nadal co kilka miesięcy obserwujemy kolejne fale zachorowań i hospitalizacji. Sytuacji tej nie udało się zmienić, pomimo powszechnie dostępnych, bezpłatnych i aktualizowanych szczepień przeciw SARS-CoV-2. Szczepienia te są obecnie zalecane we wszystkich grupach wiekowych, a zasady ich prowadzenia zostały również uwzględnione w prezentowanych rekomendacjach. Oddzielne rozdziały poświęcono odrębnościom obrazu klinicznego COVID-19 u dzieci oraz następstwom COVID-19, pomimo że są one znacznie rzadziej obserwowane w czasach dominacji wariantu Omikron. Jednocześnie ograniczono do minimum zakres informacji związanych z intensywną terapią, wychodząc z założenia że wiąże się to z wysoce specjalistycznymi, często technicznymi procedurami opisanymi w stosownych rekomendacjach.

Należy zauważyć, że konieczne mogą okazać się dalsze aktualizacje zaleceń, jeśli pojawią się nowsze i klinicznie istotne (sub)linie lub dostępne staną się nowe opcje terapeutyczne lub profilaktyczne. Należy także podkreślić, że niektóre z przedstawionych zaleceń mogą nie być w pełni zgodne z praktykami w innych systemach opieki zdrowotnej lub ich wdrożenie może być utrudnione przez ograniczoną dostępność zasobów.

2. Charakterystyka SARS-CoV-2: Etiologia, ewolucja molekularna i patogeneza

SARS-CoV-2, należący do rodziny Coronaviridae (podrodzaj Sarbecovirus), wykazuje ~96% zgodności genetycznej z wirusem RaTG13 pochodzącym od nietoperzy [8]. Transmisja do człowieka prawdopodobnie obejmowała gospodarza pośredniego, co sugerują analizy filogenetyczne, a jego powstanie przypisuje się naturalnym procesom ewolucyjnym [9-11]. Hipoteza laboratoryjnego wycieku pozostaje niepotwierdzona. Dokładne odtworzenie zdarzeń, które doprowadziły do skoku międzygatunkowego i adaptacji wirusa do organizmu człowieka może jednak nigdy nie być w pełni możliwe [12].

Od początku rozprzestrzeniania się SARS-CoV-2 w populacji ludzkiej, wirus podlega zmienności genetycznej oraz zjawiskom selekcji pod wpływem różnych czynników, takich jak środowisko organizmu gospodarza i oddziaływanie czynników zewnętrznych. Ewolucja SARS-CoV-2 zachodzi głównie na drodze mutacji punktowych, a także rekombinacji genetycznej i może mieć wpływ na epidemiologię, przebieg kliniczny zakażeń, skuteczność szczepień i terapii. Kluczowym (choć nie jedynym) obszarem zmienności jest gen kodujący białko kolca (S), odpowiadające za wiązanie z receptorem powierzchniowym komórki. Monitoring zmienności wirusa pozwala na wydzielanie jego filogenetycznych linii rozwojowych i identyfikację poszczególnych subwariantów. Najważniejsze z nich są klasyfikowane są przez WHO w zależności od cech fenotypowych i rozpoznanego ryzyka epidemiologicznego do trzech grup: (1) warianty pod obserwacją (VUM, ang. Variants under Monitoring), (2) warianty zainteresowania (VOI, ang. Variants of Interest) oraz (3) warianty budzące obawy (VOC, ang. Variants of Concern). W przeszłości do grupy VOC, najważniejszej z punktu widzenia zdrowia publicznego, zaliczano warianty Alfa (B.1.1.7), Beta (B.1.351), Delta (B.1.617.2), Gamma (P1) i Omikron (B.1.1.529). Jednak rozprzestrzenienie się Omikronu od listopada 2021 r. doprowadziło do wyparcia wszystkich innych linii rozwojowych SARS-CoV-2. W rezultacie w 2023 r. zaktualizowano klasyfikację WHO, tak by do poszczególnych grup, VUM, VOC i VOC, zaliczać tylko i wyłącznie poszczególne sublinie Omikronu. Takie podejście pozwala lepiej rozumieć wpływ zachodzącej zmienności Omikronu na jego istotność epidemiologiczną i kliniczną oraz modyfikować, w zależności od potrzeb, działania profilaktyczne i terapeutyczne [13].

Niezależnie od wariantu, SARS-CoV-2 uwalniany w trakcie kaszlu lub kichania w formie bioaerozolu szerzy się głównie drogą kropelkowo-powietrzną, rzadziej kropelkowo-pyłową. Do zakażenia komórek dochodzi w wyniku połączenia białka S wirusa z funkcjonalnym receptorem komórkowym, enzymem konwertującym angiotensynę II (ACE-2). Następnie, w zależności od miejsca proteolitycznej aktywacji białka S, wirus wnika do wnętrza komórek bezpośrednio poprzez fuzję błony komórkowej i wirusowej lub na drodze endocytozy. Silną ekspresję receptora ACE-2 zaobserwowano w komórkach nabłonka górnych dróg oddechowych, nabłonka pęcherzykowego typu 1 i 2 i śródbłonka płuc, ale również gruczołów śluzowych cewkowo-pęcherzowych górnej części przełyku, w enterocytach jelita krętego i okrężnicy, w nerkach, sercu, trzustce oraz, co wydaje się być kluczowym elementem patogenezy, w śródbłonku naczyń tętniczych i żylnych oraz komórkach mięśni gładkich naczyń tętniczych [14]. Mnogość lokalizacji ACE-2 wpływa na złożony i ogólnoustrojowy obraz kliniczny zakażenia. Wykryto też alternatywne receptory dla SARS-CoV-2, m.in. ASGR1, KREMEN1, receptor histaminowy 1, czy neuropilina 1 [15-17].

Po wniknięciu wirusa dochodzi do replikacji i uszkodzenia komórek, co może wywoływać miejscowy stan zapalny i zaburzenia funkcji narządów. Różnorodność i stopień nasilenia objawów wynika od poziomu replikacji wirusa u poszczególnych pacjentów oraz różnic w sprawności układu odporności. U niektórych pacjentów (np. osoby starsze, obciążone wielochorobowością, osoby młode z pierwotnym/wtórnym deficytem odporności) nadmierna odpowiedź immunologiczna związana z nasilonym

stanem zapalnym, w tym burzą cytokinową, prowadzi do uszkodzeń wielonarządowych, zespołu ostrej niewydolności oddechowej oraz dysfunkcji śródbłonka, co sprzyja powstawaniu zakrzepów i innych powikłań naczyniowych [18, 19].

Pojawienie się i rozprzestrzenienie linii rozwojowej Omikronu przyniosło istotne zmiany w patogenezie SARS-CoV-2, wynikające z kumulacji różnych mutacji w jego genomie, w tym rekordowej ich liczby w obrębie genu kodującego białko S. Omikron, w przeciwieństwie do poprzedzających go linii rozwojowych wirusa, wydaje się preferować endosomalną drogę wejścia do komórek, w mniejszym stopniu przyczyniając się do powstawania fuzyjnych struktur komórkowych, częściej infekując górne drogi oddechowe, z mniejszym tropizmem do tkanek płucnych [20-22]. Tłumaczy to jego potencjalnie łagodniejszy przebieg kliniczny w porównaniu z wcześniejszymi wariantami, takimi jak Delta. Jednak zwiększona transmisyjność Omikronu i zdolność ucieczki immunologicznej przyczynia się do szybkiego rozprzestrzenienia się jego subwariantów, stwarzając wyzwania w kontrolowaniu szerzenia zakażeń, zwiększając ryzyko reinfekcji i wymuszając konieczność aktualizacji składu antygenowego szczepionek.

3. Obraz kliniczny choroby

Obraz kliniczny COVID-19 uległ zmianie od czasu pojawienia się pierwszych przypadków choroby u ludzi. Obecnie w okresie dominacji subwariantów Omikronu charakteryzujących się łagodniejszym przebiegiem, dostępności do szczepionek i leków oraz wysokiego poziomu odporności populacji wynikającej z przebytych zakażeń i/lub szczepień, ogólne ryzyko wystąpienia ciężkiej postaci choroby jest znacznie mniejsze. Jednak nadal zakażenie SARS-CoV-2 w różnych grupach pacjentów, w tym powyżej 60 roku życia, zwłaszcza z chorobami współistniejącymi, oraz kobiet w ciąży jest przyczyną hospitalizacji i zgonów [23, 24]. Okres inkubacji choroby wynosi 3-4 dni i jest zwykle dłuższy niż w innych wirusowych infekcjach dróg oddechowych, z którymi wymaga różnicowania [25]. Transmisja po 6-7 dniach od wystąpienia objawów jest mało prawdopodobna, choć możliwa od osób z niedoborami odporności, u których wiremia może utrzymywać się dłużej [26]. Odsetek bezobjawowych zakażeń w ogólnej populacji szacowany jest na około 32%, jest wyższy u osób zaszczepionych i poniżej 20 roku życia [27]. Cechą charakterystyczną COVID-19 jest różnorodność objawów, w większości niespecyficznych, oraz ich zmienne nasilenie, na podstawie którego można wyróżnić cztery stadia choroby.

Stadium 1 (łagodne, bez zapalenia płuc) obejmuje pacjentów bezobjawowych lub z łagodnymi objawami i saturacją tlenem (SpO2) ≥95% na powietrzu atmosferycznym. Najczęstsze objawy to: gorączka, kaszel, zmęczenie, trudności w oddychaniu, bóle głowy, gardła, mięśni, zatkany nos, katar, nudności, wymioty, biegunka [23, 28, 29]. Choroba obejmuje przede wszystkim górne drogi oddechowe, a badaniem przedmiotowym i obrazowym nie stwierdza się cech zapalenia płuc. Anosmia lub ageuzja są rzadko raportowane [23, 30]. U kobiet w ciąży gorączka, duszność, kaszel, bóle mięśni występują rzadziej niż u niециężarnych pacjentek [24]. U starszych osób brak gorączki i zespoły geriatryczne są najczęstszymi nietypowymi objawami choroby [31]. W większości zakażeń SARS-CoV-2 choroba kończy się w tym stadium.

W stadium 2 (umiarkowane, z ewentualnym zapaleniem płuc) zaawansowania choroby pacjenci mają kliniczne i obrazowe objawy śródmiąższowego zapalenia płuc z SpO2 poniżej 95% na powietrzu atmosferycznym. Pacjenci w tym stadium mogą wymagać tlenoterapii i z tego powodu, zwłaszcza przy obecności czynników ryzyka uzasadniona jest hospitalizacja, co przy właściwym postępowaniu pozwala w większości przypadków uniknąć dalszej progresji choroby.

Stadium 3 (ciężkie, zapalenie płuc z uszkodzeniami wielonarządowymi i burzą cytokinową) to ciężkie zapalenie płuc, któremu towarzyszy co najmniej jeden z objawów: częstość oddechów powyżej 30/min, ciężka niewydolność oddechowa, SpO2<90% na powietrzu atmosferycznym. W tym stadium często pojawiają się powikłania psychiatryczne oraz neurologiczne obejmujące szerokie spektrum od omdleń do udarów [32, 33]. Dochodzi także do uszkodzenia serca manifestującego się jego zapaleniem, zawałem z uniesieniem odcinka ST, arytmiami i zapaleniem osierdzia [34, 35]. Biomarkerem ciężkiego przebiegu choroby jest podwyższone stężenie troponiny w surowicy krwi [36]. Wykazano, że ostre uszkodzenie mięśnia sercowego u pacjentów, którzy przeżyli nie postępuje a rokowanie średnioterminowe jest korzystne [37]. U niemal wszystkich chorych w tym stadium występują zaburzenia krzepnięcia świadczące o wykrzepianiu śródnaczyniowym i zagrożeniu zakrzepowo-zatorowym, które przynajmniej częściowo tłumaczą uszkodzenia wielonarządowe.

Stadium 4 (krytyczne, z zespołem ostrej niewydolności oddechowej (ARDS) i niewydolnością wielonarządową) rozwija się zwłaszcza u osób obciążonych czynnikami ryzyka (wiek >60 lat, otyłość, cukrzyca, choroby nowotworowe, przewlekła niewydolność serca, przewlekła niewydolność oddechowa, przewlekła niewydolność nerek, niedobory odporności oraz immunosupresja). W tym stadium obserwujemy ostrą niewydolność oddechową, której towarzyszy wstrząs septyczny i/lub dysfunkcja wielonarządowa, w tym ostre uszkodzenie nerek i wątroby [28, 29]. Istnieje wysokie ryzyko tętniczej i żylnej choroby zakrzepowo-zatorowej, które utrzymuje się przez co najmniej rok po zakażeniu [38]. Współistniejące infekcje bakteryjne są powszechne u pacjentów hospitalizowanych w OIT i stanowią, obok ARDS, odmy i niewydolności wielonarządowej, główną przyczynę zgonów [39].

4. Diagnostyka laboratoryjna

Standardem potwierdzającym zakażenie SARS-CoV-2 pozostaje niezmiennie wykrycie antygenów wirusowych lub materiału genetycznego wirusa w próbkach pobranych z nosogardzieli. Co ważne, w dobie odporności hybrydowej w populacji (uprzednie przechorowania, szczepienia lub ich kombinacja) oraz krążących subwariantów Omikronu, czułość testów antygenowych lub genetycznych zmienia się w kolejnych dniach od początku objawów choroby, będąc najwyższą pomiędzy 3-4 dniem. Przykładowo dla testów antygenowych 30.0%-60.0% pierwszego dnia, 59.2%-74.8% trzeciego dnia i 80.0%-93.3% czwartego dnia objawów z gwałtownym zmniejszeniem po 5 dniu objawów [40].

4.1. Testy Antygenowe

W dobie krążących subwariantów Omikronu i odporności hybrydowej testy antygenowe pozostają wysoko czułe, pozwalając na wykrycie większości zakażeń SARS-CoV-2, co przekłada się na stosunkowo tanią diagnostykę i ułatwienie ograniczania ryzyka transmisji. Czułość testowania jest niższa u osób bezobjawowych [41]. Aktualnie testy antygenowe powinny spełniać następujące wymogi [42]:

- posiadają certyfikat CE,
- skuteczność kliniczna została potwierdzona na próbkach pobranych z jamy nosowej, jamy ustno-gardłowej lub jamy nosowo-gardłowej,
- czułość >80% w badaniach na pacjentach objawowych w pierwszych siedmiu dniach od wystąpienia objawów lub u osób bezobjawowych, u których zakażenie SARS CoV-2 zostało potwierdzone za pomocą testów molekularnych,

lub

- czułość >90% dla osób, u których wykryto materiał genetyczny SARS CoV-2 z progiem detekcji (cycle threshold, CT) <25, odzwierciedlającym wysoki ładunek wirusa.

W użyciu są aktualnie również multipleksowe testy antygenowe, pozwalające na jednoczesne wykrycie antygenów SARS CoV-2, grypy (często z różnicowaniem grypy A i B), RSV oraz innych wirusów związanych z zakażeniami górnych dróg oddechowych. Czułość tych testów jest wyższa w przypadku osób z wysokim ładunkiem wirusowym, ale pozwalają one na szybkie prowadzenie diagnostyki różnicowej [43].

4.2. Testy Molecularne

Testy molekularne wykrywające materiał genetyczny wirusa (NAAT – nucleic acid amplification testing) pozostają testami o wysokiej czułości i swoistości. Testowanie ma wyższą czułość w porównaniu z testowaniem antygenowych dla wykrycia zakażenia w pierwszych dniach choroby lub u osób bezobjawowych. Należy zwrócić uwagę, że wartość CT może być wartościowym wskaźnikiem ładunku wirusa [44]. Wartości CT <25 odzwierciedlają wysokie ładunki SARS CoV-2, podczas gdy wartości >35 oznaczają resztkową obecność materiału genetycznego i często nie są związane z aktywnym zakażeniem. Co ważne, materiał genetyczny SARS-CoV-2 może być wykrywalny długotrwale przez ponad 3 miesiące, a w przypadku osób z niedoborem odporności nawet do 9 miesięcy i nie wiąże się to z replikacją wirusa [45].

4.3. Testy Serologiczne

W dobie odporności hybrydowej, gdy większość populacji była eksponowana na SARS-CoV-2 lub szczepiona, testy serologiczne nie mają praktycznego zastosowania klinicznego. Miana przeciwciał serologicznych spadają po 6-9 miesiącach od zakażenia lub immunizacji SARS CoV-2, przy czym większe spadki występują w populacjach wrażliwych, zwłaszcza u osób starszych oraz osób w immunosupresji lub z odpornością osłabioną z innych powodów [46]. Ponadto standardowe testy serologiczne nie oceniają swoistej odpowiedzi na pojawiające się nowe warianty SARS CoV-2. Klinicznie przeciwciała anty-SARS CoV-2 można analizować u osób z zaburzeniami odporności, które nie wytwarzają przeciwciał, aby ocenić odpowiedź po szczepieniu lub po chorobie.

5. Leczenie

Postępowanie terapeutyczne w COVID-19 zależy od stadium klinicznego choroby, stanu pacjenta, oraz ryzyka ciężkiego przebiegu choroby. Schemat zalecanego postępowania terapeutycznego przedstawiono na rycinie 1, a szczegóły znajdują się w tabeli 1. Przebieg choroby można podzielić na cztery stadia, które różnicują sposób postępowania terapeutycznego (rycina 1).

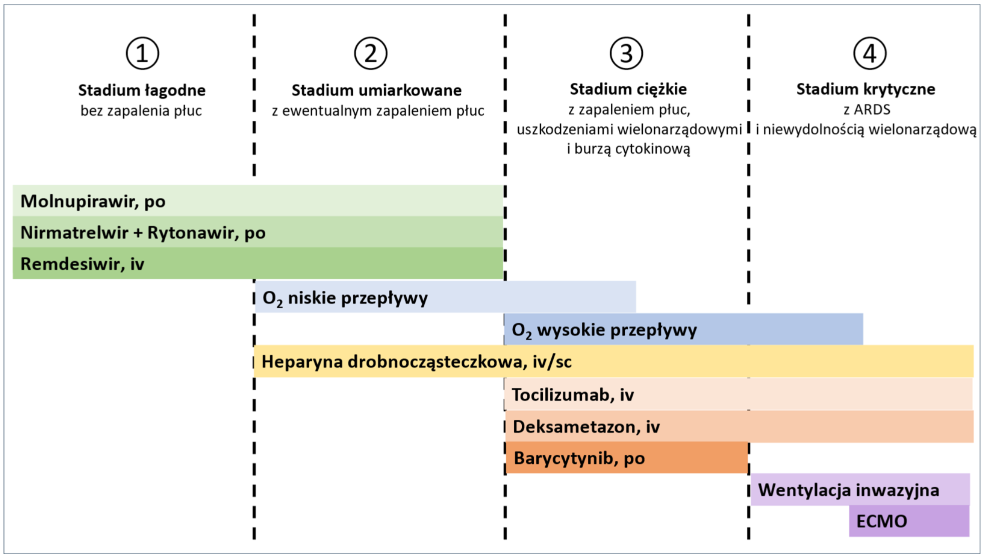

Rycina 1. Schemat zalecanej terapii COVID-19.

5.1. Stadium 1 (łagodne, bez zapalenia płuc)

Zdecydowana większość chorych przechodzi zakażenie SARS-CoV-2 bezobjawowo lub skąpoobjawowo. Odsetek takich chorych zmienia się w zależności od charakterystyki dominującego wariantu wirusa. Łagodny przebieg choroby może jednak poprzedzać progresję do cięższego przebiegu choroby, zwłaszcza u osób należących do grup ryzyka. Pacjenci w stadium 1 nie wymagają hospitalizacji, ale powinni pozostawać pod opieką lekarza podstawowej opieki zdrowotnej, który dokona oceny stanu ogólnego, SpO2 oraz czynników ryzyka progresji do ciężkiej postaci COVID-19, do których zaliczamy: wiek >60 lat, otyłość, cukrzycę, choroby nowotworowe, przewlekłą niewydolność serca, przewlekłą niewydolność oddechową, przewlekłą niewydolność nerek, niedobory odporności oraz immunosupresję. Chorzy należący do którejkolwiek z tych grup, niezależnie od stanu klinicznego, powinni jak najwcześniej otrzymać terapię przeciwwirusową molnupirawirem, nirmatrelwirem/rytonawirem, lub remdesiwirem (kolejność alfabetyczna, nie wskazuje na pierwszeństwo wyboru) w celu zmniejszenia ryzyka progresji choroby i konieczności hospitalizacji. Leczenie powinno być zastosowane do 5. doby od wystąpienia objawów [47-57]. U chorych w immunosupresji, wynikającej ze współistniejącej choroby lub stosowanej terapii, czas włączenia leczenia przeciwwirusowego można wydłużyć do 10 dni od wystąpienia objawów COVID-19 z racji dłuższego okresu replikacji wirusa. Podczas terapii lekami przeciwwirusowymi [58-60] i bezpośrednio po jej zakończeniu u kobiet w wieku rozrodczym zalecana jest skuteczna antykoncepcja, aczkolwiek stosowanie remdesiwiru jest dopuszczalne w 2. i 3. trymestrze ciąży oraz u kobiet karmiących piersią. Planując stosowanie nirmatrelwiru/rytonawiru, należy ocenić ryzyko interakcji z innymi lekami przyjmowanymi przez pacjenta, korzystając z narzędzi internetowych [61]. Szczegóły dawkowania i przeciwwskazania do stosowania leków przeciwwirusowych przedstawiono w tabeli 1. U chorych nieobciążonych czynnikami ryzyka, z łagodnymi objawami infekcji dróg oddechowych, takimi jak gorączka, ból gardła, głowy, mięśni, nieżyt nosa, leczenie przeciwwirusowe można rozważyć, kierując się stanem klinicznym pacjenta, sytuacją epidemiologiczną i dostępnością leków. W ramach leczenia objawowego pacjenci mogą wymagać stosowania leków przeciwgorączkowych (paracetamol) i przeciwkaszlowych. Budezonid stosowany wziewnie może być wykorzystywany jako leczenie wspomagające łagodzące przebieg choroby [62, 63]. U chorych w stadium 1. glikokortykosteroidy stosowane systemowo są przeciwwskazane

ze względu na ich działanie immunosupresyjne, mogące nasilać i wydłużać czas replikacji wirusa, a przez to pogarszać rokowanie [64]. U chorych przewlekle unieruchomionych i z innymi wskazaniami do profilaktyki przeciwzakrzepowej niezwiązanymi z COVID-19, a zwłaszcza u chorych z czynnikami ryzyka wystąpienia zakrzepicy żył głębokich i/lub zatoru płucnego, wskazane jest stosowanie heparyny drobnocząsteczkowej w dawkach profilaktycznych [65, 66]. Brak jest dowodów na korzyści wynikające ze stosowania antybiotyków w tym stadium choroby, dlatego antybiotykoterapię można rozważyć wyłącznie w przypadku rozpoznania lub podejrzenia infekcji bakteryjnej dróg oddechowych wynikającego z obrazu klinicznego, badań mikrobiologicznych i/lub obrazowych [67-69].

5.2. Stadium 2 (umiarkowane, z ewentualnym zapaleniem płuc)

Pogorszenie stanu klinicznego, zwłaszcza z wystąpieniem duszności wymagającej tlenoterapii i obniżeniem SpO2 poniżej 95% stanowi wskazanie do hospitalizacji. Pacjenci obciążeni współchorobowością, u których w przebiegu zakażenia SARS-CoV-2 nastąpiło pogorszenie przebiegu choroby zasadniczej, powinni być skierowani do szpitala do oddziału właściwego dla leczenia tego schorzenia, nawet przy braku wskazań do tlenoterapii. W takich przypadkach terapia przeciwwirusowa, jeżeli została rozpoczęta w ramach podstawowej opieki zdrowotnej, powinna być kontynuowana. Zwykle w tej fazie choroby wystarczająca jest tlenoterapia niskoprzepływowa, nieprzekraczająca 15 l/min. Profilaktyczne dawki heparyny drobnocząsteczkowej stanowią element standardowego postępowania u chorych hospitalizowanych, z opcją zwiększenia w uzasadnionych przypadkach do dawek terapeutycznych. Jeżeli od wystąpienia objawów COVID-19 nie upłynęła 5. doba (w immunosupresji 10. doba) należy rozpocząć leczenie przeciwwirusowe [58-60]. W przypadku chorych hospitalizowanych należy stosować doustnie molnupirawir lub nirmatrelwir/rytonawir albo dożylnie remdesiwir (kolejność alfabetyczna, nie wskazuje na pierwszeństwo wyboru) [49-52, 58-60]. Terapia przeciwwirusowa w warunkach szpitalnych jest zalecana również u chorych niewymagających tlenoterapii, w celu zapobiegania progresji związanej z czynnikami ryzyka lub ograniczenia szerzenia się zakażenia wśród innych pacjentów i personelu. Remdesiwir może być podawany niezależnie od stosowanej tlenoterapii, a nirmatrelwir/rytonawir przed jej zastosowaniem. Dołączenie glikokortykosteroidów do leków przeciwwirusowych w tym stadium choroby nie przynosi korzyści, a poprzez działanie immunosupresyjne może nasilać lub przedłużać replikację wirusa. Jednak w przypadku braku poprawy klinicznej pomimo stosowania terapii przeciwwirusowej, można rozważyć zastosowanie deksametazonu w drugim tygodniu choroby [64]. W przypadku rozpoznania lub podejrzenia zakażenia bakteryjnego wynikającego z obrazu klinicznego, badań mikrobiologicznych i/lub obrazowych można rozważyć zastosowanie antybiotyków [69]. Ograniczenia w stosowaniu leków oraz szczegóły dawkowania przedstawiono omawiając stadium 1 i w tabeli 1.

5.3. Stadium 3 (ciężkie, zapalenie płuc z uszkodzeniami wielonarządowymi i burzą cytokinową)

Pogorszenie stanu klinicznego może wystąpić na początku 2. tygodnia choroby z narastaniem duszności i redukcją SpO2 poniżej 90% powoduje u części chorych konieczność zastosowania tlenoterapii wysokoprzepływowej do 60 l/min. Jednocześnie może to świadczyć o rozpoczynającej się burzy cytokinowej, czego potwierdzeniem będzie wzrost stężenia interleukiny 6 (IL-6) we krwi. Przekroczenie stężenia 100 pg/ml uzasadnia podanie tocilizumabu, przeciwciała monoklonalnego skierowanego przeciw receptorowi IL-6, co istotnie zmniejsza ryzyko wentylacji mechanicznej i zgonu [70-76]. Tocilizumab należy podawać we wlewie dożylnym w dawce zależnej od masy ciała. W przypadku braku efektu, kolejny wlew można podać po 8-24 godzinach, lecz nie

udowodniono korzyści wynikających z podania dwóch dawek w porównaniu z pojedynczą dawką [76]. Zastosowanie glikokortykosteroidów dożylnie w dawce dziennej nie przekraczającej 6 mg deksametazonu jest wskazane, gdy pogorszenie stanu klinicznego przebiega bez wzrostu stężenia IL-6 lub gdy zastosowanie tocilizumabu nie przyniosło efektu [72, 77]. Stosowanie wyższych dawek deksametazonu pogarsza rokowanie [78, 79]. Alternatywą dla tocilizumabu może być podawany doustnie inhibitor kinazy janusowej - barycytynib, którego skuteczność wykazano zwłaszcza u chorych wymagających tlenoterapii wysokoprzepływowej [80-83]. Szczegóły dawkowania i przeciwwskazania leków zalecanych w stadium 3 przedstawiono w tabeli 1. Terapia skojarzona wyżej wymienionymi lekami nie jest zalecana ze względu na brak danych z badań klinicznych. Na tym etapie choroby pacjenci powinni nadal otrzymywać heparynę drobnocząsteczkową. Stosowanie leków przeciwwirusowych w tym stadium choroby nie jest zasadne, za wyjątkiem chorych w immunosupresji, u których istnieje ryzyko przedłużonego utrzymywania się wiremii SARS-CoV-2. Antybiotykoterapia jest uzasadniona tylko w przypadku wysokiego prawdopodobieństwa zakażenia bakteryjnego wynikającego z obrazu klinicznego, badań mikrobiologicznych i/lub obrazowych [69].

5.4. Stadium 4 (krytyczne, z zespołem ostrej niewydolności oddechowej (ARDS) i niewydolnością wielonarządową)

Pogorszenie stanu pacjenta pomimo stosowanego leczenia i stosowanej tlenoterapii wysokoprzepływowej oznacza zwykle ARDS. Chory w tym stadium wymaga wentylacji inwazyjnej płuc, a także zastosowania glikokortykosteroidów [64, 84]. Jeśli pacjent nie otrzymał wcześniej tocilizumabu lub baricytynibu to można rozważyć podanie któregoś z tych immunomodulatorów [76, 80]. Nie ma uzasadnienia do rozpoczynania lub kontynuowania leczenia przeciwwirusowego u chorych wentylowanych inwazyjnie, za wyjątkiem pacjentów pozostających w immunosupresji wynikającej z wcześniejszej choroby lub stosowanego leczenia. Mechaniczną wentylację inwazyjną należy stosować w oddziałach intensywnej terapii, zgodnie ze ustalonymi standardami postępowania [85, 86]. Stosowanie żylno-żylnego pozaustrojowego natleniania membranowego (VV ECMO) można rozważyć tylko u wybranych chorych i powinno być realizowane zgodnie z międzynarodowymi wytycznymi w ośrodkach posiadających odpowiednie doświadczenie i możliwości techniczne [86, 87].

Tabela 1. Zalecane postępowanie terapeutyczne z uwzględnieniem leczenia podstawowego i wspomagającego w poszczególnych stadaich COVID-19.

1 stadium (łagodne, bez zapalenia płuc)<br>• ***1 tydz. choroby***<br>• ***SpO_2 ≥95%***<br>• ***podstawowa opieka zdrowotna***	
**Leczenie podstawowe**	**Leczenie wspomagające**
**Leki przeciwwirusowe** (kolejność alfabetyczna, nie wskazuje na pierwszeństwo wyboru). Rozpoczęcie terapii przeciwwirusowej do 5. doby od wystąpienia objawów, a do 10 dni w stanach immunosupresji. Zalecane u chorych z ryzykiem ciężkiego przebiegu COVID-19*, do rozważenia u pozostałych chorych.<br>• **Molnupirawir**, tylko dorośli, doustnie, 2 razy dziennie 800 mg przez 5 dni. Przeciwwskazany u kobiet w ciąży i karmiących [58].<br>LUB	• spoczynek,<br>• nawodnienie doustne,<br>• Budezonid wziewnie, 2 x 800 µg dziennie [62],<br>• leki przeciwgorączkowe (paracetamol, ibuprofen itd.),

• **Nirmatrelwir/rytonawir**, tylko dorośli, doustnie 2 razy dziennie 300/100mg przez 5 dni. Należy sprawdzić interakcje międzylekowe [61]. Przeciwwskazany:<br>- u kobiet w ciąży i karmiących<br>- w niewydolności wątroby Child-Pugh C,<br>- przy eGFR<30 ml/min, a przy eGFR 30-60 ml/min redukcja dawki do 150/100mg, [59].<br>LUB<br>• **Remdesiwir**, dorośli i dzieci z masą ciała >40 kg, dożylnie, 1 raz dziennie przez 3 dni, w 1. dobie 200 mg, w 2. i 3. dobie 100 mg. Przeciwwskazany u kobiet w pierwszym trymestrze ciąży; dopuszczalny w 2 i 3 trymestrze oraz u kobiet karmiących piersią [60].	• leki przeciwkaszlowe w przypadku uporczywego kaszlu,<br>• heparyna drobnocząsteczkowa u chorych przewlekle unieruchomionych i z innymi wskazaniami do profilaktyki przeciwzakrzepowej niezwiązanymi z COVID-19.
**Uwagi:**<br>• Glikokortykosteroidy ogólnoustrojowe przeciwwskazane.<br>• Antybiotykoterapia wyłącznie w przypadku rozpoznania lub uzasadnionego podejrzenia nakładającej się infekcji bakteryjnej dróg oddechowych.	
2 stadium (umiarkowane z zapaleniem płuc)<br>• 1-2 tydz. choroby<br>• SpO_2 <95%<br>• hospitalizacja	
**Leczenie podstawowe**	**Leczenie wspomagające**
**Tlenoterapia:** niskoprzepływowa, do 15 l/min<br>**Leki przeciwzakrzepowe:** Heparyna drobnocząsteczkowa w dawce profilaktycznej, którą można zwiększyć w uzasadnionych przypadkach.<br>**Leki przeciwwirusowe** (kolejność alfabetyczna, nie wskazuje na pierwszeństwo wyboru). Rozpoczęcie terapii przeciwwirusowej do 5. dobyod wystąpienia objawów, a do 10 dni w stanach immunosupresji.<br>• **Molnupirawir**, tylko dorośli, doustnie, 2 razy dziennie 800 mg przez 5 dni. Przeciwwskazany u kobiet w ciąży i karmiących [58].<br>LUB<br>• **Nirmatrelwir/rytonawir**, tylko dorośli, doustnie 2 razy dziennie 300/100mg przez 5 dni. Należy sprawdzi interakcje międzylekowe [61]. Przeciwwskazany:<br>- u kobiet w ciąży i karmiących<br>- w niewydolności wątroby Child-Pugh C,<br>- przy eGFR<30 ml/min, a przy eGFR 30-60 ml/min redukcja dawki 150/100 mg, [59]<br>LUB<br>• **Remdesiwir**:<br>- dorośli i dzieci z masą ciała >40 kg, dożylnie, 1 raz dziennie przez 5-10 dni, w 1. dobie 200 mg, kolejnych dobach 100 mg.<br>- dzieci >4 tygodnia z masą ciała 3-40 kg, dożylnie, 1 raz dziennie do 10 dni, w 1. dobie 5mg/kg, w kolejnych dobach 2,5 mg/kg.<br>Przeciwwskazany u kobiet w pierwszym trymestrze ciąży; dopuszczalny w 2 i 3 trymestrze oraz u kobiet karmiących piersią [60].	• leczenie objawowe,<br>• nawodnienie doustne lub dożylne
**Uwagi:**<br>• Glikokortykosteroidy do rozważenia w 2 tygodniu choroby, ale tylko w przypadku braku poprawy klinicznej pomimo	

<table>
<tr><td colspan="2">stosowania leków przeciwwirusowych i tlenoterapii; u dorosłych deksametazon doustnie lub dożylnie 4-8 mg/d, u dzieci 0.1-0.15 mg/kg dziennie (maksymalnie 6 mg) nie dłużej niż przez 10 dni.
• Antybiotykoterapia wyłącznie w przypadku ropoznania lub uzasadnionego podejrzenia nakładającej się infekcji bakteryjnej dróg oddechowych.</td></tr>
<tr><td colspan="2">3 stadium (ciężkie, zapalenie płuc z uszkodzeniem wielonarządowym i burzą cytokinową)
• 2 tydz. Choroby
• SpO2 <90%
• hospitalizacja</td></tr>
<tr><th>Leczenie podstawowe</th><th>Leczenie wspomagające</th></tr>
<tr><td>Tlenoterapia: wysokoprzepływowa, do 60 l/min.
Leki przeciwzakrzepowe: Heparyna drobnocząsteczkowa w dawce profilaktycznej, którą można zwiększyć w uzasadnionych przypadkach.
Glikokortykosteroidy: Deksametazon podawany dożylnie dorosłym w dawce dziennej 6 mg, a u dzieci 0.1-0.15 mg/kg dziennie (maksymalnie 6 mg) nie dłużej niż przez 10 dni, [78, 79].
Leki immunomodulacyjne:
• Tocilizumab, dorośli, u których stężenie IL 6 przekracza 100 pg/ml, 60-minutowy wlew dożylny, 8 mg/kg, a w przypadku braku poprawy druga dawka może być powtórzona po 8–24 godz. Przeciwwskazania:
- liczba neutrofili <1000/µl,
- płytek krwi <50 tys./µl,
- aktywność aminotransferazy alaninowej >10-krotnej górnej granicy normy [76].
LUB
• Barycytynib, dorośli doustnie, raz dziennie 4 mg, do 14 dni. Przeciwwskazany:
- neutropenia <500/µL,
- limfopenia <200/µL,
- eGFR<15 mL/min lub dializowani [80].</td><td>• leczenie objawowe,
• nawodnienie dożylne</td></tr>
<tr><td colspan="2">Uwagi:
Antybiotykoterapia wyłącznie w przypadku rozpoznania lub uzasadnionego podejrzenia nakładającej się infekcji bakteryjnej dróg oddechowych.</td></tr>
<tr><td colspan="2">4 stadium (krytyczne z ARDS i niewydolnością wielonarządową)
• 2-3 tydz. Choroby
• konieczność wentylacji inwazyjnej
• oddział intensywnej terapii</td></tr>
<tr><th>Leczenie podstawowe</th><th>Leczenie wspomagające</th></tr>
<tr><td>Terapia oddechowa:
• Tlenoterapia wysokoprzepływowa: do 60 l/min
LUB
• Wentylacja inwazyjna
LUB
• Pozaustrojowa żylno-żylna przezbłonowa oksygenacja (VV ECMO) u wybranych chorych,</td><td>• symptomatic treatment
• intravenous hydration</td></tr>
</table>

**Leki przeciwzakrzepowe:** Heparyna drobnocząsteczkowa w dawce profilaktycznej, którą można zwiększyć w uzasadnionych przypadkach.<br>**Glikokortykosteroidy:** Deksametazon, dożylnie, u dorosłych dawka dzienna 12 mg, a u dzieci 0.1-0.15 mg/kg dziennie (maksymalnie 6 mg) nie dłużej niż przez 10 dni. Jeśli deksametazon nie jest dostepny, można podawać inne glikokortykoidy w równoważnych dawkach.<br>LUB/I<br>**Leki immunomodulacyjne:**<br>• **Tocilizumab**, dorośli, jeśli nie podano wcześniej to w pierwszej dobie wentylacji mechanicznej 60-minutowy wlew dożylny, 8 mg/kg. Przeciwwskazania:<br>- neutropenia <1000/μL,<br>- płytki krwi <50 thousand/μL,<br>- aktywność aminotransferazy alaninowej >10-krotnej górnej granicy normy [76].<br>LUB<br>• **Barycytynib**, dorośli doustnie (dożołądkowo), raz dziennie 4 mg, do 14 dni. Przeciwwskazany:<br>- neutropenia <500/μl,<br>- limfopenia <200/μl,<br>- eGFR<15 mL/min lub dializowani [80].	
**Uwagi:**<br>Antybiotykoterapia wyłącznie w przypadku rozpoznania lub uzasadnionego podejrzenia nakładającej się infekcji bakteryjnej dróg oddechowych.	

* wiek >60 lat, otyłość, cukrzyca, choroba nowotworowa, przewlekła niewydolność serca, przewlekła niewydolność oddechowa, przewlekła niewydolność nerek, niedobory odporności, immunosupresja

6. Terapie o niepotwierdzonej skuteczności

W początkowym okresie pandemii rozważano stosowanie licznych leków w terapii COVID-19, które miały przede wszystkim wykazywać działanie przeciwwirusowe. W przypadku niektórych terapii skuteczność była oceniana w badaniach klinicznych, chociaż jedynie dla pojedynczych były to randomizowane badania kontrolowane. Oczywiście znikoma siła dowodów dla badań obserwacyjnych lub serii przypadków nie pozwala na umieszczanie takich terapii w rekomendacjach. Dla poniżej przedstawionych leków dotychczas nie potwierdzono skuteczności, wykazano ich nieskuteczność lub niebezpieczeństwo ich stosowania.

6.1. Leki przeciwwirusowe/przeciwinfekcyjne

- Fawipirawir (główny artykuł wspierający został wycofany).
- Oseltamiwir i zanamiwir [88].
- Amantadyna i rymantadyna [89].
- Leki antyretrowirusowe (stosowane w zakażeniach HIV) [90].
- Iwermektyna [91].
- Fluwoksamina [92].
- Interferony.
- Immunoglobulina dożylna oraz immunoglobulina specyficzna przeciwko SARS-CoV-2 [93].

- Przeciwciała monoklonalne anty-SARS-CoV-2 – bamlaniwimab/etesewimab, casirivimab/imdewimab, tixagevimab/cilgavimab brak skuteczności wobec wariantu Omikron SARS-CoV-2 [94].
- Osocze ozdrowieńców [95].

6.2. Leki przeciwzapalne

- Niesteroidowe leki przeciwzapalne (NLPZ) mogą być stosowane w leczeniu objawowym w dawkach terapeutycznych i przez krótki czas [96].
- Anakinra, pomimo początkowego zalecania jej stosowania w stadium 3, ostatecznie nie wykazała skuteczności w badaniach klinicznych [97].
- Glikortykosteroidy są przeciwwskazane w 1 stadium choroby.

6.3. Inne leki

- Metformina [98].
- Suplementy diety, w tym witaminy C i D oraz cynk [99].
- Azytromycyna i inne antybiotyki powinny być stosowane jedynie w przypadku współistniejącej infekcji bakteryjnej [100].
- Chlorochina i hydroksychlorochina [101].

W przypadku infekcji u pacjentów przyjmujących wcześniej leki immunosupresyjne lub jakiekolwiek leki z powodu chorób współistniejących zaleca się utrzymanie tych terapii w trakcie COVID-19.

7. Zespół pokowidowy

Określenie „zespół pokowidowy" (ang. post-COVID syndrome) odnosi się do objawów utrzymujących się lub zaburzeń funkcji narządów występujących po ostrej fazie COVID-19 [102]. W październiku 2021 r. WHO opublikowała definicję przypadku zespołu pokowidowego, która wskazuje, że można go rozpoznać u chorych z prawdopodobnym lub potwierdzonym zakażeniem SARS-CoV-2. Jego objawy występują zwykle po upływie 3 miesięcy od wystąpienia COVID-19, trwają co najmniej 2 miesiące i nie są związane z inną diagnozą [103]. W nowej klasyfikacji ICD-10-CM zespół pokowidowy uzyskał kod U09.9.

Częstość występowania zespołu waha się od 10% do 60% i zależy od wielu czynników, takich jak płeć, obecność chorób przewlekłych czy też deficytów odporności, ciężkość przebiegu zakażenia, stosowanego leczenia przeciwwirusowego w fazie ostrej oraz szczepienia anty-SARS-CoV-2. Metaanalizy pokazują, że do najczęstszych objawów należą zmęczenie (32-57%), zaburzenia snu (10-55%), duszność (17-38%), osłabienie (8%-56%), bóle w klatce piersiowej (11-24%), bóle głowy (9-27%) oraz stawów (7-24%) [104]. Pediatryczny wieloukładowy zespół zapalny (PIMS/MIS-C) jest odrębnym stanem ujawniającym się po COVID-19 u dzieci i młodych dorosłych, spełniających określone kryteria diagnostyczne [105]. Wyniki polskiego badania prospektywnego SILCOV-19 (The Silesian Complications of COVID-19 Database) potwierdzają, że najczęściej występującymi objawami zespołu pokowidowego są zmęczenie, duszność, kołatania serca oraz zaburzenia węchu i smaku [106].

Dolegliwości kardiologiczne dotyczą ok 15% chorych po COVID-19 i manifestują się najczęściej poprzez bóle w klatce piersiowej (OR 4,0), kołatanie serca (OR 3,4) lub nadciśnienie (OR 1,7) [107]. Jednym ze zdefiniowanych fenotypów po COVID-19 jest zespół przewlekłego zmęczenia (ME/CFS). Zespół pokowidowy jest częstszy u kobiet [108], u chorych z zaburzeniami odporności oraz chorobami przewlekłymi takimi jak astma oskrzelowa, przewlekła niewydolność oddechowa, cukrzyca typu 2, niewydolność serca, czy przewlekła choroba nerek [109]. Objawy zespołu pokowidowego zwykle cofają

się z czasem, chociaż wykazano, że większość chorych z tą diagnozą nadal zgłasza objawy w drugim roku od rozpoznania [110].

Nie ma dowodów na rekomendowanie rutynowych badań przesiewowych u osób niewykazujących objawów. Diagnostyka powinna być ukierunkowana na zgłaszane dolegliwości [111]. Kluczowe znaczenie ma edukacja chorych, w szczególności w zakresie objawów zespołu pokowidowego. Obiektywnymi metodami oceny choroby płuc są: skala duszności Borga, pulsoksymetria domowa, test 6-minutowego marszu (6MWT), testy czynnościowe płuc. W przypadku objawów kardiologicznych stosuje się 24-godzinne monitorowanie metodą Holtera i echokardiogram. Obecnie brak jest zaleceń co do rutynowej oceny parametrów krzepnięcia po COVID-19.

W rekonwalescencji po COVID-19 ważną rolę odgrywa odpoczynek, aktywność fizyczna i rehabilitacja oddechowa. Nie ma dowodów na celowość stosowania rutynowej profilaktyki przeciwzakrzepowej, chociaż chorzy z grup wysokiego ryzyka mogą wymagać stosowania leków przeciwkrzepliwych nawet do 30 dni po wypisie zgodnie z zaleceniami ogólnymi [112, 113]. W badaniach randomizowanych wykazano korzystny wpływ rehabilitacji oddechowej w połączeniu z aktywnością aerobową [114] oraz kontrolowaną utratą masy ciała u osób z nadwagą [115] na zmniejszenie się objawów zespołu pokowidowego.

Zespół pokowidowy rozwija się rzadziej u osób zaszczepionych przeciw COVID-19 zarówno przed, jak i po zachorowaniu [116], a także u tych, które w ostrej fazie infekcji otrzymały celowane leczenie przeciwwirusowe [117]. Obecnie te działania stanowią kluczowy element strategii walki z zespołem pokowidowym. W badaniach randomizowanych, nie potwierdzono efektywności klinicznej niektórych leków postulowanych jako skutecznych w leczeniu zespołu pokowidowego (m. in. losartanu, imatynibu), dalsze badania pozostają w toku.

8. Odrębności kliniczne i terapeutyczne u dzieci

Początkowo niskie wskaźniki zakażeń u dzieci znacznie wzrosły w okresie dominacji wariantu Omikron. Według danych z Wielkiej Brytanii w okresie listopad–grudzień 2021 r. wskaźniki seropozytywności wynosiły 37% u dzieci w wieku 1-4 lat, 54% u dzieci w wieku 5-11 lat, 78% u dzieci w wieku 12-15 lat i 87% u dzieci w wieku 16-17 lat. Do września 2022 r. seroprewalencja wzrosła odpowiednio do 93%, 98%, 99% i 99% [118]. W polskim badaniu oceniającym seroprewalencję przeciwciał IgG przeciw SARS-CoV-2 u 686 dzieci hospitalizowanych z innych przyczyn niż COVID-19 od 1 czerwca 2021 r. do 30 kwietnia 2022 r. wykazano obecność tych przeciwciał u 57% hospitalizowanych oraz wzrost odsetka do 87,5% w trakcie czwartej i piątej fali COVID-19 (warianty Delta i Omikron). Zdecydowana większość rodziców badanych dzieci nie miała wiedzy o zakażeniu COVID-19 u swoich podopiecznych, co może świadczyć o zakażeniu bezobjawowym lub łagodnym przebiegu choroby [119].

Dzieci zazwyczaj wykazują łagodniejsze objawy lub pozostają bezobjawowe. Do mechanizmów patofizjologicznych odpowiadających za łagodny przebieg choroby u dzieci zalicza się: niższą ekspresję receptora ACE2, słabsze powinowactwo wiązania między receptorami ACE2 a białkami kolca, silną wrodzoną i nabytą odpowiedź immunologiczną, fizjologicznie wysoki odsetek limfocytów u młodszych dzieci, mniejszą liczbę chorób współistniejących oraz znaczną odporność krzyżową ze względu na wcześniejsze narażenie na zakażenia innymi koronawirusami. U dzieci z COVID-19 obserwowano wyższe poziomy IgM, IgG i interferonu oraz niższe IL-6 i IL-10 [120]. Obraz kliniczny COVID-19 był związany z wiekiem i dominującym wariantem SARS-CoV-2 w momencie zakażenia. Ryzyko hospitalizacji w dzieciństwie z powodu ciężkiego przebiegu COVID-19 zawsze pozostawało bardzo niskie, a długość pobytu w szpitalu u większości dzieci wynosiła 1-2 dni. Wyższe ryzyko hospitalizacji dotyczyło

niemowląt oraz dzieci z chorobami współistniejącymi. Ryzyko hospitalizacji było niższe w przypadku wariantu Omikron w porównaniu z wcześniejszymi wariantami SARS-CoV-2 [121].

Analiza przebiegu COVID-19 u 1283 polskich dzieci, obejmująca okres od 1 marca 2020 r. do 31 grudnia 2020 r. wykazała, że najczęstszym objawem była gorączka (46%). U najmłodszych dzieci częściej obserwowano gorączkę, nieżyt nosa i biegunkę natomiast nastolatki częściej skarżyły się na ból głowy, ból gardła, zaburzenia węchu i smaku oraz osłabienie. Jedna piąta pacjentów pozostała bezobjawowa. Zapalenie płuc zdiagnozowano u 12% pacjentów, częściej u młodszych dzieci. Podczas drugiej fali zakażeni pacjenci byli młodsi i wymagali dłuższej hospitalizacji [122]. COVID-19 u niemowląt objawiał się łagodną infekcją przewodu pokarmowego lub układu oddechowego, ale obserwowano również zapalenie płuc ze spadkiem saturacji wymagające tlenoterapii [123].

Ryzyko PIMS (ang. paediatric inflammatory multisystem syndrome, pediatryczny wieloukładowy zespół zapalny) po zakażeniu SARS-CoV-2 obniżyło się z 0,038% podczas dominacji wariantu Alfa do 0,026% podczas dominacji wariantu Delta. Obecnie ryzyko PIMS jest <0,01%, co może być spowodowane zmianami genetycznymi w antygenach powierzchniowych SARS-CoV-2, odpowiedzialnych za hiperzapalną odpowiedź immunologiczną [124]. Biorąc pod uwagę, że prawie wszystkie dzieci mają przeciwciała przeciwko SARS-CoV-2, głównie w następstwie infekcji, ale także szczepienia, spodziewany jest niewielki odsetek cięższych przebiegów choroby, w tym zapalenia mięśnia sercowego i PIMS [125].

Większość pacjentów pediatrycznych z COVID-19 wymaga jedynie leczenia objawowego. Leki przeciwwirusowe (remdesiwir) i glikokortykosteroidy są zarezerwowane dla ciężkich przypadków, szczególnie u pacjentów wymagających tlenoterapii [126]. Istotnymi czynnikami ryzyka zespołu pokowidowego u dzieci były: wiek >12 lat, choroby współistniejące oraz płeć żeńska. Szczepienie wiązało się z niższym ryzykiem zespołu pokowidowego u starszych dzieci i niższym ryzykiem ponownych zakażeń [127].

Pandemia COVID-19 znacząco wpłynęła na zdrowie psychiczne dzieci z powodu ograniczeń wynikających z dystansu społecznego i zdalnego nauczania. U dzieci i młodzieży obserwuje się nasilenie zaburzeń lękowych i depresji [128]. Uważa się, że wzrost częstości występowania otyłości w populacji pediatrycznej w ciągu ostatniej dekady został wzmocniony przez pandemię [129]. Niewątpliwie następstwa immunologiczne wywołane pandemią COVID-19 mają związek z obserwowanymi zmianami w obrazie epidemiologiczno-klinicznym wielu chorób zakaźnych u dzieci.

9. Szczepienia

Szczepienia przeciw COVID-19 pozostają kluczowym elementem profilaktyki ciężkiego przebiegu choroby, hospitalizacji oraz zgonów z jej powodu [130-132]. Zalecenia dotyczące szczepień dostosowuje się do sytuacji epidemiologicznej, ewolucji wirusa SARS-CoV-2 i dostępności szczepionek. Przewiduje się regularne wprowadzanie nowych szczepionek przeciwko COVID-19, dostosowanych do krążących wariantów wirusa [130-132]. Szczepionki te będą uwzględniały uaktualnione rekomendacje WHO oraz Europejskiej Agencji Leków i zostaną opracowane w oparciu o technologię mRNA lub podjednostkową [133]. Poniżej przedstawiono najważniejsze informacje i zalecenia szczepień w Polsce aktualne na dzień publikacji niniejszych rekomendacji.

9.1. Dostępne szczepionki

W sezonie 2024/2025 w Unii Europejskiej zarejestrowane są następujące szczepionki przeciw COVID-19:

Szczepionki mRNA:

- Comirnaty (Pfizer-BioNTech)
- Spikevax (Moderna)

Szczepionki białkowe podjednostkowe (niedostępne w Polsce):

- Nuvaxovid (Novavax) – zawierająca rekombinowane białko S oraz adiuwant Matrix-M
- Bimervax (Hipra) – zawierająca rekombinowane białko S oraz adiuwant SQBA

Wszystkie wymienione szczepionki są monowalentne, skierowane przeciw dominującemu podwariantowi JN.1 SARS-CoV-2 [130, 134-136].

9.2. Grupy docelowe szczepień

Szczepienia przeciw COVID-19 są zalecane przede wszystkim osobom z grup podwyższonego ryzyka ciężkiego przebiegu choroby, w tym:

- osobom w wieku ≥60 lat,
- osobom z chorobami przewlekłymi, w tym cukrzycą, chorobami płuc, nerek, układu sercowo-naczyniowego, otyłością (BMI ≥25), zaburzeniami neurorozwojowymi, czynną chorobą nowotworową lub w stanie immunosupresji (wynikającej z choroby lub leczenia),
- osobom przebywającym w placówkach opieki długoterminowej
- kobietom w ciąży, ze względu na redukcję powikłań COVID-19 wśród noworodków
- osobom pracującym w placówkach opieki zdrowotnej lub opieki długoterminowej
- dzieciom w wieku od 6 miesięcy do 11 lat, szczególnie z chorobami przewlekłymi [130, 137-139].

9.3. Schemat szczepień

Obecnie zaleca się uproszczony schemat szczepień przeciwko COVID-19:

- Osobom w wieku ≥12 lat bez istotnych czynników ryzyka: podawanie pojedynczej dawki szczepionki raz w roku, niezależnie od dotychczasowej historii szczepień i zachorowań na COVID-19. Odstęp od poprzedniej dawki lub przebytego zakażenia powinien wynosić co najmniej 3 miesiące [131, 132, 137].
- Osobom z ciężkim niedoborem odporności: zaleca się podawanie szczepionki co 6 miesięcy, w minimalnym odstępie 2 miesięcy między dawkami . Jeśli jest możliwe, optymalnie ≥2 tygodni przed rozpoczęciem/kontynuacją leczenia immunosupresyjnego.
- Powtórzenie szczepienia zaleca się pacjentom szczepionym przed lub w trakcie leczenia przeszczepem komórek krwiotwórczych lub terapią komórkami CAR T ≥ 3 miesiące po zabiegu. Powtórzenie szczepienia należy rozważyć u pacjentów szczepionych przeciw COVID-19 podczas leczenia terapiami zubożającymi komórki B – zaleca się szczepienie 6 miesięcy po terapii. W przypadku planowanej terapii zubożającej komórki B szczepienia przeciwko COVID-19 należy podać 4 tygodnie przed jej rozpoczęciem lub wznowieniem [131].
- Dzieciom w wieku od 6 miesięcy do 11 lat: szczepionym po raz pierwszy zalecamy podanie 2 dawek w odstępie 4 tygodni, a w przypadku dzieci z ciężkim niedoborem odporności - 3 dawek (dwie pierwsze w odstępie 4 tygodni, trzecia po 2 miesiącach od drugiej dawki). Jeśli jest możliwe,

optymalnie ≥2 tygodni przed rozpoczęciem/kontynuacją leczenia immunosupresyjnego. Dzieci w immunosupresji powinny otrzymywać kolejne dawki szczepionki co 6 miesięcy.

- Bezpieczne i zalecane jest podawanie szczepionki przeciwko COVID-19 równocześnie z inaktywowaną szczepionką przeciw grypie lub szczepionką przeciwko pneumokokom (na jednej wizycie), a także pozostałymi szczepionkami podawanymi rutynowo.
- Szczepionki przeciw COVID-19 można podawać w dowolnym odstępie od innych szczepionek (w tym zalecanych w ciąży) z wyjątkiem szczepionki przeciw ospie małpiej (MPox), wówczas należy zachować odstęp minimum 4 tygodni.
- Szczepionki podaje się domięśniowo, zalecamy kontynuowanie szczepień w miarę możliwości szczepionką tego samego wytwórcy. Nie zaleca się stosowania szczepionek o nieaktualnym składzie.

9.4. Przeciwwskazania i sytuacje wymagające ostrożności

Przeciwwskazaniem do szczepienia jest:

- Ciężka reakcja alergiczna (np. anafilaksja) po podaniu poprzedniej dawki szczepionki lub na którykolwiek jej składnik,
- Ostra choroba z gorączką lub zaostrzenie choroby przewlekłej – szczepienie należy odroczyć do czasu ustąpienia objawów.

Szczególną ostrożność należy zachować u osób, u których po poprzedniej dawce szczepionki wystąpiło:

- Zapalenie mięśnia sercowego lub osierdzia,
- Wieloukładowy zespół zapalny (MIS-C u dzieci albo MIS-A u dorosłych).
- Zalecamy monitorowanie wszystkich szczepionych przez minimum 15 minut po szczepieniu.

9.5. Bezpieczeństwo szczepionek

Szczepionki przeciw COVID-19 są bezpieczne i na ogół dobrze tolerowane. Najczęstsze niepożądane odczyny poszczepienne (NOP) to:

- ból, zaczerwienienie lub obrzęk w miejscu wstrzyknięcia,
- zmęczenie, ból głowy, mięśni lub stawów, gorączka.

Rzadko obserwuje się poważne NOP, takie jak zapalenie mięśnia sercowego lub osierdzia, które występują głównie u młodych mężczyzn w wieku 12–39 lat. Ryzyko to jest jednak bardzo niskie, a korzyści ze szczepienia znacznie przewyższają potencjalne ryzyko [132, 140, 141]. Rzadkie zdarzenia zakrzepowo-zatorowe znane jako immunozakrzepowa trombocytopenia wywołana szczepionką (VITT), w której uczestniczą przeciwciała przeciw czynnikowi płytkowemu 4 (PF4), zostały w większości powiązane ze szczepionkami wektorowymi adenowirusowymi. Zdarzenia te są rzadko zgłaszane po szczepionkach mRNA, co wskazuje na fundamentalne różnice w mechanizmach immunologicznych wyzwalanych przez te odrębne platformy szczepionkowe.

9.6. Organizacja szczepień

Szczepienia przeciw COVID-19 powinny być finansowane przez Ministerstwo Zdrowia, dostępne bezpłatnie dla jak najszerszej populacji i realizowane w:

- podstawowej opiece zdrowotnej (lekarze rodzinni),
- aptekach ogólnodostępnych,
- szpitalach.

Szczepienia przeciw COVID-19 cechują się wysoką skutecznością w zapobieganiu hospitalizacjom i zgonom z powodu COVID-19. Szczepionki mają dobry profil bezpieczeństwa i są dobrze tolerowane, a możliwe niepożądane odczyny poszczepienne mają zazwyczaj łagodny i przemijający charakter [132, 140, 141].

10. Profilaktyka przedekspozycyjna

Mimo zdecydowanie łagodniejszego przebiegu zakażenia aktualnie dominującymi wariantami SARS-CoV-2, profilaktykę przedekspozycyjnę należy rozważyć u osób z umiarkowanym lub ciężkim upośledzeniem odporności lub obarczonych wielochorobowością, zagrożonych ciężkim przebiegiem COVID-19, zwłaszcza jeśli z jakichś powodów nie zostały zaszczepione. Najskuteczniejszą formą profilaktyki swoistej są szczepienia, które omówiono wcześniej. Przeciwciała monoklonalne stały się nieprzydatne w profilaktyce przedekspozycyjnej wraz z dominacją wariantu Omikron, ale mogą być rozważane u osób zagrożonych ciężkim przebiegiem COVID-19 pod warunkiem, że pojawią się dowody naukowe na skuteczność konkretnego leku wobec aktualnie dominującego wariantu SARS-CoV-2 [142, 143]. Jednak w związku ze zmiennością wariantów wirusa przydatność leków opartych na przeciwciałach monoklonalnych bardzo szybko wygasa, a najnowszym przykładem jest pemivibart, który uzyskał autoryzację amerykańskiej Agencji Żywności i Leków (FDA) w marcu 2024 r., która jednak nie ulega dalszemu rozszerzeniu [144, 145]. Skuteczność profilaktyki farmakologicznej opartej na stosowaniu nirmatrelwiru/rytonawiru nie została potwierdzona, choć wykazano zmniejszenie ryzyka nawrotu zakażenia wariantem Omikron [146]. W ramach profilaktyki nieswoistej biernej zaleca się unikania kontaktu z osobami zakażonymi, noszenie masek ochronnych FFP2 lub FFP3, unikanie zgromadzeń w zamkniętych pomieszczeniach, odpowiednią wentylację pomieszczeń, częste mycie rąk, a w skrajnych przypadkach izolacja do czasu ustąpienia przyczyn i skutków niedoboru odporności. Należy zauważyć, że pacjenci z COVID-19, którzy w przeszłości wykonywali ćwiczenia oporowe i wytrzymałościowe, są mniej narażeni na hospitalizację i śmiertelność, ale mechanizm tego zjawiska wymaga dalszych badań [147]

Supplemetary Materials: Niniejszy dokument jest suplementem do wersji artykułuw w języku angielskim

Wkład autorów: Konceptualizacja, R.F.; metodologia, R.F.; walidacja, analiza formalna i badanie, R.F.; J.J.; D.K.; E.K.; M.Par.; M.Paw.; A.P.; P.R.; K.S.; K.T.; D.Z.M.; zasoby, R.F.; gromadzenie danych, R.F.; pisanie — przygotowanie oryginalnego projektu, R.F.; J.J.; D.K.; E.K.; M.Par.; M.Paw.; A.P.; P.R.; K.S.; K.T.; D.Z.M.; pisanie — recenzja i edycja, R.F.; P.R.; D.Z.M.; wizualizacja, P.R.; nadzór, R.F.; administrowanie projektem, R.F.; Wszyscy autorzy przeczytali i zaakceptowali opublikowaną wersję manuskryptu.

Finansowanie: Badania te nie otrzymały żadnego zewnętrznego dofinansowania.

Oświadczenie instytucji: Nie dotyczy.

Świadoma zgoda: Nie dotyczy.
Oświadczenie o dostępności danych: Dla potrzeb tego artykułu nie stworzono nowych danych.

Konflikt interesu: granty, doradztwo, lub/i honoraria - R.F.: Gilead, Moderna, MSD, Novavax, Pfizer, Roche; J.J.: Gilead, Moderna, MSD, Pfizer, Roche; D.K.: nie zgłasza konfliktu interesów; E.K.: Pfizer, Moderna, Novavax, MSD; M.Par.: Pfizer, Roche; M.Paw.: Gilead, MSD, Pfizer, Roche; A.P.: Gilead, Pfizer; P.R.: Moderna, Pfizer; K.S.: Gilead, MSD, Roche, Red Hill Biopharma; K.T.: AstraZeneca, Gilead, Pfizer, Roche; D.Z.M.: Gil-ead, Pfizer, Roche.

Piśmiennictwo

1. WHO COVID-19 dashboard. https://data.who.int/dashboards/covid19/cases. (accessed, 2 March 2025)
2. Flisiak, R.; Horban, A.; Jaroszewicz, J.; Kozielewicz, D.; Pawłowska, M.; Parczewski, M.; Piekarska, A.; Simon, K.; Tomasiewicz, K.; Zarębska-Michaluk D. Management of SARS-CoV-2 infection: recommendations of the Polish Association of Epidemiologists and Infectiologists as of March 31, 2020. *Pol. Arch. Intern. Med.* **2020**, *130*, 352-357.
3. Flisiak, R.; Horban, A.; Jaroszewicz, J.; Kozielewicz, D.; Pawłowska, M.; Parczewski, M.; Piekarska, A.; Simon, K.; Tomasiewicz, K.; Zarębska-Michaluk D. Management of SARS-CoV-2 infection: recommendations of the Polish Association of Epidemiologists and Infectiologists. Annex no. 1 as of June 8, 2020. *Pol. Arch. Intern. Med*. **2020**, *130*, 557-558.
4. Flisiak, R.; Parczewski, M.; Horban, A.; Jaroszewicz, J.; Kozielewicz, D.; Pawłowska, M.; Piekarska, A.; Simon, K.; Tomasiewicz, K.; Zarębska-Michaluk D. Management of SARS-CoV-2 infection: recommendations of the Polish Association of Epidemiologists and Infectiologists. Annex no. 2 as of October 13, 2020. *Pol. Arch. Intern. Med*. **2020**, *130*, 915-918.
5. Flisiak, R.; Horban, A.; Jaroszewicz, J.; Kozielewicz, D.; Mastalerz-Migas, A.; Owczuk, R.; Parczewski, M.; Pawłowska, M.; Piekarska, A.; Simon, K.; Tomasiewicz, K.; Zarębska-Michaluk D. Management of SARS-CoV-2 infection: recommendations of the Polish Association of Epidemiologists and Infectiologists as of April 26, 2021. *Pol. Arch. Intern. Med*. **2021**, *131*, 487-496.
6. Flisiak, R.; Horban, A.; Jaroszewicz, J.; Kozielewicz, D.; Mastalerz-Migas, A.; Owczuk, R.; Parczewski, M.; Pawłowska, M.; Piekarska, A.; Simon, K.; Tomasiewicz, K.; Zarębska-Michaluk D. Diagnosis and therapy of SARS-CoV-2 infection: recommendations of the Polish Association of Epidemiologists and Infectiologists as of November 12, 2021. Annex no. 1 to the Recommendations of April 26, 2021. *Pol. Arch. Intern. Med.* **2021**, *131*, 16140.
7. Flisiak, R.; Horban, A.; Jaroszewicz, J.; Kozielewicz, D.; Mastalerz-Migas, A.; Owczuk, R.; Parczewski, M.; Pawłowska, M.; Piekarska, A.; Simon, K.; Tomasiewicz, K.; Zarębska-Michaluk D. Management of SARS-CoV-2 infection: recommendations of the Polish Association of Epidemiologists and Infectiologists as of February 23, 2022. *Pol. Arch. Intern. Med*. **2022**, *132*, 16230.
8. Zhou, P.; Yang, X.-L.; Wang, X.-G.; Hu, B.; Zhang, L.; Zhang, W.; Si, H.-R.; Zhu, Y.; Li, B.; Huang, C.-L.; et al. A Pneumonia Outbreak Associated with a New Coronavirus of Probable Bat Origin. *Nature* **2020**, *579*, 270–273.
9. Andersen, K.G.; Rambaut, A.; Lipkin, W.I.; Holmes, E.C.; Garry, R.F. The Proximal Origin of SARS-CoV-2. *Nat. Med.* **2020**, *26*, 450–452.
10. Boni, M.F.; Lemey, P.; Jiang, X.; Lam, T.T.-Y.; Perry, B.W.; Castoe, T.A.; Rambaut, A.; Robertson, D.L. Evolutionary Origins of the SARS-CoV-2 Sarbecovirus Lineage Responsible for the COVID-19 Pandemic. *Nat. Microbiol.* **2020**, *5*, 1408–1417.
11. Li, L.-L.; Wang, J.-L.; Ma, X.-H.; Sun, X.-M.; Li, J.-S.; Yang, X.-F.; Shi, W.-F.; Duan, Z.-J. A Novel SARS-CoV-2 Related Coronavirus with Complex Recombination Isolated from Bats in Yunnan Province, China. *Emerg. Microbes Infect.* **2021**, *10*, 1683–1690.
12. Searching for SARS-CoV-2 Origins: Confidence versus Evidence. *Lancet Microbe* 2023, *4*, e200.
13. Statement on the Update of WHO's Working Definitions and Tracking System for SARS-CoV-2 Variants of Concern and Variants of Interest. Available online: https://www.who.int/news/item/16-03-2023-statement-on-the-update-of-who-s-working-definitions-and-tracking-system-for-sars-cov-2-variants-of-concern-and-variants-of-interest (accessed, 2 march 2025).
14. Jackson, C.B.; Farzan, M.; Chen, B.; Choe, H. Mechanisms of SARS-CoV-2 Entry into Cells. *Nat. Rev. Mol. Cell Biol.* **2022**, *23*, 3–20.

15. Cantuti-Castelvetri, L.; Ojha, R.; Pedro, L.D.; Djannatian, M.; Franz, J.; Kuivanen, S.; van der Meer, F.; Kallio, K.; Kaya, T.; Anastasina, M.; Smura, T.; Levanov, L.; Szirovicza, L.; Tobi, A.; Kallio-Kokko, H.; Österlund, P.; Joensuu, M.; Meunier, F.A.; Butcher, S.J.; Winkler, M.S.; Mollenhauer, B.; Helenius, A.; Gokce, O.; Teesalu, T.; Hepojoki, J.; Vapalahti, O.; Stadelmann, C.; Balistreri, G.; Simons, M. Neuropilin-1 Facilitates SARS-CoV-2 Cell Entry and Infectivity. *Science* **2020**, *370*, 856–860.
16. Gu, Y.; Cao, J.; Zhang, X.; Gao, H.; Wang, Y.; Wang, J.; He, J.; Jiang, X.; Zhang, J.; Shen, G.; Yang, J.; Zheng, X.; Hu, G.; Zhu, Y.; Du, S.; Zhu, Y.; Zhang, R.; Xu, J.; Lan, F.; Qu, D.; Xu, G.; Zhao, Y.; Gao, D.; Xie, Y.; Luo, M.; Lu, Z. Receptome Profiling Identifies KREMEN1 and ASGR1 as Alternative Functional Receptors of SARS-CoV-2. *Cell Res.* **2022**, *32*, 24–37.
17. Yu, F.; Liu, X.; Ou, H.; Li, X.; Liu, R.; Lv, X.; Xiao, S.; Hu, M.; Liang, T.; Chen, T.; Wei, X.; Zhang, Z.; Liu, S.; Liu, H.; Zhu, Y.; Liu, G.; Tu, T.; Li, P.; Zhang, H.; Pan, T.; Ma, X. The Histamine Receptor H1 Acts as an Alternative Receptor for SARS-CoV-2. *MBio* **2024**, *15*, e0108824.
18. Lamers, M.`M.; Haagmans, B.L. SARS-CoV-2 Pathogenesis. *Nat. Rev. Microbiol.* **2022**, *20*, 270–284.
19. Zaidi, A.K.; Singh, R.B.; A A Rizvi, S.; Dehgani-Mobaraki, P.; Palladino, N. COVID-19 Pathogenesis. *Prog. Mol. Biol. Transl. Sci.* **2024**, *202*, 67–112.
20. Willett, B.J.; Grove, J.; MacLean, O.A.; Wilkie, C.; De Lorenzo, G.; Furnon, W.; Cantoni, D.; Scott, S.; Logan, N.; Ashraf, S.; Manali, M.; Szemiel, A.; Cowton, V.; Vink, E.; Harvey W.T.; Davis, C.; Asamaphan, P.; Smollett, K.; Tong, L.; Orlon, R.; Hughes, J.; Holland, P.; Silva, V.; Pascall, D.J.; Puxty, K.; da Silva Filipe, A.; Yebra, G.; Shaaban, S.; Holden M.T.G.; Pinto, R.M.; Gunson, R.; Templeton, K.; Murcia, P.R.; Patel, A.H.; Klenerman, P.; Dunachie, S. SARS-CoV-2 Omicron Is an Immune Escape Variant with an Altered Cell Entry Pathway. *Nat. Microbiol.* **2022**, *7*, 1161–1179.
21. Meng, B.; Abdullahi, A.; Ferreira, I.A.T.M.; Goonawardane, N.; Saito, A.; Kimura, I.; Yamasoba, D.; Gerber, P.P.; Fatihi, S.; Rathore, S.; Zepeda, S.K.; Papa, G.; Kemp, S.A; Ikeda, T.; Toyoda, M.; Tan, T.S.; Kuramochi, J.; Mitsunaga, S.; Ueno, T.; Shirakawa, K.; Takaori-Kondo, A.; Brevini, T.; Mallery, D.L.; Charles, O.J.; Bowen, J.E.; Joshi, A.; Walls, A.C.; Jackson, L.; Martin, D.; Smith, K.G.C; Bradley, J.; Briggs, J.A.G.; Choi, J.; Madissoon, E.; Meyer, K.B.; Mlcochova, P.; Ceron-Gutierrez, L.; Doffinger, R.; Teichmann, S.A.; Fisher, A.J.; Pizzuto, M.S.; de Marco, A.; Corti, D.; Hosmillo, M.; Lee, J.H.; James L.C.; Thukral, L.; Veesler, D.; Sigal, A.; Sampaziotis, F.; Goodfellow, I.G.; Matheson1, N.J.; Sato, K.; Gupta, R.K. Altered TMPRSS2 Usage by SARS-CoV-2 Omicron Impacts Infectivity and Fusogenicity. *Nature* **2022**, *603*, 706–714.
22. Hui, K.P.Y.; Ho, J.C.W.; Cheung, M.-C.; Ng, K.-C.; Ching, R.H.H.; Lai, K.-L.; Kam, T.T.; Gu, H.; Sit, K.-Y.; Hsin, M.K.Y.; Au, T.W.K.; Poon, L.L.M.; Peiris, M.; Nicholls, J.M.; Chan M.C.W. SARS-CoV-2 Omicron Variant Replication in Human Bronchus and Lung Ex Vivo. *Nature* **2022**, *603*, 715–720.
23. Flisiak, R.; Zarębska-Michaluk, D.; Dobrowolska, K.; Rorat, M.; Rogalska, M.; Kryńska, J.A.; Moniuszko-Malinowska, A.; Czupryna, P.; Kozielewicz, D.; Jaroszewicz, J.; Sikorska, K.; Bednarska, A.; Piekarska, A.; Rzymski, P.. Change in the Clinical Picture of Hospitalized Patients with COVID-19 between the Early and Late Period of Dominance of the Omicron SARS-CoV-2 Variant. *J Clin Med*. **2023**, *12*, 5572.
24. Allotey, J.; Stallings, E.; Bonet, M.; Yap, M.; Chatterjee, S.; Kew, T.; Zhou, D.; Coomar, D.; Sheikh, J.; Lawson, H.; Ansari, K.; Attarde, S.; Littmoden, M.; Banjoko, A.; Barry, K.; Akande, O.; Sambamoorthi, D.; van Wely, M.; van Leeuwen, E.; Kostova, E.; Kunst, H.; Khalil, A.; Tiberi, S.; Brizuela, V.; Broutet, N.; Kara, E.; Kim, C.R.; Thorson, A.; Escuriet, R.; Gottlieb, S.; Tong, V.T.; Ellington, S.; Oladapo, O.T.; Mofenson, L.; Zamora, J.; Thangaratinam, S. Clinical manifestations, risk factors, and maternal and perinatal outcomes of coronavirus disease 2019 in pregnancy: living systematic review and meta-analysis. *BMJ* **2020**, *370,* m3320.
25. Wu, Y.; Kang, L.; Guo, Z.; Liu, J.; Liu, M.; Liang W. Incubation Period of COVID-19 Caused by Unique SARS-CoV-2 Strains: A Systematic Review and Meta-analysis. *JAMA Netw Open* **2022**, *5*, e2228008.

26. Rahmani, A.; Dini, G.; Leso, V.; Montecucco, A.; Kusznir Vitturi, B.; Iavicoli, I.; Durando, P. Duration of SARS-CoV-2 shedding and infectivity in the working age population: a systemic review and meta-analysis. *Med. Lav* **2022**, *113*, e2022014.
27. Shang, W.; Kang, L.; Cao, G.; Wang, Y.; Gao, P.; Liu, J.; Liu, M. Percentage of Asymptomatic Infections among SARS-CoV-2 Omicron Variant-Positive Individuals: A Systematic Review and Meta-Analysis. *Vaccines (Basel)* **2022**, *10*, 1049.
28. WHO. Clinical management of COVID-19: Living guideline, 18 August 2023. https://www.who.int/publications/i/item/WHO-2019-nCoV-clinical-2023.2 (accessed, 2 March 2025).
29. Clinical characteristics of COVID-19. European Center for Disease Prevention and Control. Updated: 30 May 2023. https://www.ecdc.europa.eu/en/covid-19/latest-evidence/clinical (accessed, 2 March 2025).
30. Coelho, D.H.; Reiter, E.R.; French, E.; Costanzo, R.M. Decreasing Incidence of Chemosensory Changes by COVID-19 Variant. *Otolaryngol. Head Neck Surg*. **2022**, *168*, 704-706.
31. Tavares, J.; Figueiredo, D.; Passos, L.; Sobrinho, L.; Souza, E.; Pedreira, L. Atypical Presentation of COVID-19 in Older Adults: A Scoping Review. *Port. J. Public Health*. **2023**, *41*, 198-217.
32. de Freitas, R.F,; Torres, S.C.; Martín-Sánchez, F.J.; Carbó, A.V.; Lauria, G. Nunes JPL. Syncope and COVID-19 disease—a systematic review. *Auton. Neurosci*. **2021**, *235*, 102872.
33. Taquet, M.; Geddes, JR.; Husain, M.; Luciano, S.; Harrison, P.J. 6-month neurological and psychiatric outcomes in 236 379 survivors of COVID-19: a retrospective cohort study using electronic health records. *Lancet Psychiatry* **2021**, *8*: 416-427.
34. Kemerley, A.; Gupta, A.; Thirunavukkarasu, M.; Maloney, M.; Burgwardt, S.; Maulik, N. COVID-19 Associated Cardiovascular Disease-Risks, Prevention and Management: Heart at Risk Due to COVID-19. *Curr. Issues Mol. Biol.* **2024**, *46*, 1904-1920.
35. Buckley, B.J.R.; Harrison, S.L.; Fazio-Eynullayeva, E.; Underhill, P.; Lane, D.A.; Lip, G.Y.H. Prevalence and clinical outcomes of myocarditis and pericarditis in 718,365 COVID-19 patients. *Eur. J. Clin. Invest*. **2021**, *51,* e13679.
36. Bavishi, C.; Bonow, R.O.; Trivedi, V.; Abbott, J.D.; Messerli, F.H.; Bhatt, D.L. Special Article—Acute myocardial injury in patients hospitalized with COVID-19 infection: A review. *Prog. Cardiovasc. Dis*. **2020**, *63*, 682–689.
37. Shiwani, H.; Artico, J.; Moon, J.C.; Gorecka, M.; McCann, G.P.; Roditi, G.; Morrow, A.; Mangion, K.; Lukaschuk, E.; Shanmuganathan, M.; Miller, C.A.; Chiribiri, A.; Alzahir, M.; Ramirez, S.; Lin, A.; Swoboda, P.P; McDiarmid, A.K.; Sykes, R.; Singh, T.; Bucciarelli-Ducci, C.; Dawson, D.; Fontana, M.; Manisty, C.; Treibel, T.A.; Levelt, E.; Arnold, R.; Young, R.; McConnachie, A.; Neubauer, S.; Piechnik, S.K.; Davies, R.H.; Ferreira, V.M.; Dweck, M.R.; Berry, C.; Greenwood, J.P. Clinical Significance of Myocardial Injury in Patients Hospitalized for COVID-19: A Prospective, Multicenter, Cohort Study. *JACC Cardiovasc. Imaging*. **2024**, *17,* 1320-1331.
38. Knight, R.; Walker, V.; Cooper, S.J.A.; Bolton, T.; Keene, S.; Denholm, R.; Akbari, A.; Abbasizanjani, H.; Torabi, F.; Omigie, E.; Hollings, S.; North, T.l.: Toms, R.; Jiang, X.; Di Angelantonio, E.; Denaxas, S.; Thygesen, J.H.; Tomlinson, C.; Bray, B.; Smith, C.J.; Barber, M.; Khunti, K.; Smith, G.D.; Chaturvedi, N.; Sudlow, C.; Whiteley, W.N.; Wood, A.M.; Sterne, J.A.C. Association of COVID-19 with major arterial and venous thrombotic diseases: a population-wide cohort study of 48 million adults in England and Wales. *Circulation* **2022**, *146,* 892–906.
39. Liu, S.; Yu, C.; Tu, Q.; Zhang, Q.; Fu, Z.; Huang, Y.; He, C.; Yao, L. Bacterial co-infection in COVID-19: a call to stay vigilant. *PeerJ*. **2024**, *12*, e18041.
40. Frediani, J.K.; Parsons, R.; McLendon, K.B.; Westbrook, A.L.; Lam, W.; Martin, G.; Pollock, N.R. The New Normal: Delayed Peak SARS-CoV-2 Viral Loads Relative to Symptom Onset and Implications for COVID-19 Testing Programs, *Clinical Infectious Diseases* **2024**, *78*, 301–307.
41. Eyre, D.W.; Futschik, M.; Tunkel, S.; Wei, J.; Cole-Hamilton, J.; Saquib, R.; Germanacos, N.; Dodgson, A.R.; Klapper, P.E.; Sudhanva, M.; Kenny, C.; Marks, P.; Blandford, E.; Hopkins, S.; Peto, T.E.A.; Fowler, T.

Performance of antigen lateral flow devices in the UK during the alpha, delta, and omicron waves of the SARS-CoV-2 pandemic: a diagnostic and observational study. *Lancet Infectious Diseases* **2023**, *23*, 922-932.

42. EU Common list of COVID-19 antigen tests. Final bis update: 26 July 2023. https://health.ec.europa.eu/system/files/2023-12/covid-19_eu-common-list-antigen-tests_en.pdf . (accessed, 2 March 2025).
43. Murphy, C.; Mak, L.; Cheng, S.M.S.; Gigi, Y.Z.L.; Leung, K.K.Y.; Sum, N.Y.W.; Poukka, E.; Peiris J.S.M.; Cowling, B.J. Diagnostic performance of multiplex lateral flow tests in ambulatory patients with acute respiratory illness. *Diagnostic Microbiology and Infectious Disease* **2024**, *110*, 116421.
44. De Arcos-Jiménez, J.C.; Quintero-Salgado, E.; Martínez-Ayala, P.; Rosales-Chávez, G.; Damian-Negrete, R.M.; Fernández-Diaz, O.F.; Ruiz-Briseño, M.d.R.; López-Romo, R.; Vargas-Becerra, P.N.; Rodríguez-Montaño, R.; López-Yáñez, A.M.; Briseno-Ramirez, J. Population-Level SARS-CoV-2 RT-PCR Cycle Threshold Values and Their Relationships with COVID-19 Transmission and Outcome Metrics: A Time Series Analysis Across Pandemic Years. *Viruses* **2025**, *17*, 103.
45. Sepulcri, C.; Dentone, C.; Mikulska, M.; Bruzzone, B.; Lai, A.; Fenoglio, D.; Bozzano, F.; Bergna, A.; Parodi, A.; Altosole, T.; Delfino, E.; Bartalucci, G.; Orsi, A.; Di Biagio, A.; Zehender, G.; Ballerini, F.; Bonora, S.; Sette, A.; De Palma, R.; Silvestri, G.; De Maria, A.; Bassetti, M. The longest persistence of viable SARS-CoV-2 with recurrence of viremia and relapsing symptomatic COVID-19 in an immunocompromised patient—a case study. *Open Forum Infect. Dis.* **2021**, *8*, ofab217.
46. Muecksch, F., Wise, H., Templeton, K., Batchelor, B., Squires, M., McCance, K., Jarvis, L., Malloy, K., Furrie, E., Richardson, C., MacGuire, J., Godber, I., Burns, A., Mavin, S., Zhang, F., Schmidt, F., Bieniasz, P. D., Jenks, S., & Hatziioannou, T. Longitudinal variation in SARS-CoV-2 antibody levels and emergence of viral variants: a serological analysis. *Lancet Microbe* **2022**; *3*: e493–e502.
47. Jayk Bernal, A.; Gomes da Silva, M.M.; Musungaie, D.B.; Kovalchuk, E.; Gonzalez, A.; Delos Reyes, V.; Martín-Quirós, A.; Caraco, Y.; Williams-Diaz, A,; Brown, M.L.; Du, J.; Pedley, A.; Assaid, C.; Strizki, J.; Grobler, J.A.; Shamsuddin, H.H.; Tipping, R.; Wan, H.; Paschke, A.; Butterton, J.R.; Johnson, M.G.; De Anda, C. Molnupiravir for Oral Treatment of Covid-19 in Nonhospitalized Patients. *N. Engl. J. Med*. **2022**; *386*: 509-520.
48. Gottlieb, R.L.; Vaca, C.E.; Paredes, R.; Mera, J.; Webb, B.J; Perez, G.; Oguchi, G.; Ryan, P.; Nielsen, B.U.; Brown, M.; Hidalgo, A.; Sachdeva, Y.; Mittal, S.; Osiyemi, O.; Skarbinski, J.; Juneja, K.; Hyland, R.H.; Osinusi, A.; Chen, S.; Camus, G.; Abdelghany, M.; Davies, S.; Behenna-Renton, N.; Duff, F.; Marty, F.M.; Katz, M.J.; Ginde, A.A.; Brown, S.M.; Schiffer, J.T.; Hill, J.A. Early Remdesivir to Prevent Progression to Severe Covid-19 in Outpatients. *N. Engl. J. Med*. **2022**, *386*: 305-315.
49. Beigel, J.H.; Tomashek, K.M.; Dodd, L.E.; Mehta, A.K.; Zingman, B.S.; Kalil, A.C.; Hohmann, E.; Chu, H.Y.; Luetkemeyer, A.; Kline, S.; de Castilla, D.L.; Finberg, R.W.; Dierberg, K.; Tapson, V.; Hsieh, L.; Patterson, T.F.; Paredes, R.; Sweeney, D.A.; Short, W.R.; Touloumi, G.; Lye, D.C.; Ohmagari, N.; Oh, M.; Ruiz-Palacios, G.M.; Benfield, T.; Fätkenheuer, G.; Kortepeter, M.G.; Atmar, R.L.; Creech, C.B.; Lundgren, J.; Babiker, A.G.; Pett, S.; Neaton, J.D.; Burgess, T.H.; Bonnett, T.; Green, M.; Makowski, M.; Osinusi, A.; Nayak, S.; Lane, H. Remdesivir for the Treatment of Covid-19 - Final Report. *N. Engl. J. Med*. **2020**, *383*, 1813-1826.
50. Flisiak, R.; Zarębska-Michaluk, D.; Berkan-Kawińska, A.; Tudrujek-Zdunek, M.; Rogalska, M.; Piekarska, A.; Kozielewicz, D.; Kłos K.; Rorat, M.; Bolewska, B.; Szymanek-Pasternak, A.; Mazur, W.; Lorenc, B.; Podlasin, R.; Sikorska, K.; Oczko-Grzesik, B.; Iwaszkiewicz, C.; Szetela, B.; Pabjan, P.; Pawłowska, M.; Tomasiewicz, K.;

Polańska, J.; Jaroszewicz, J. Remdesivir-based therapy improved the recovery of patients with COVID-19 in the multicenter, real-world SARSTer study. *Pol. Arch. Intern. Med*. **2021**, *131*, 103-110.
51. Dobrowolska, K.; Zarębska-Michaluk, D.; Brzdęk, M.; Rzymski, P.; Rogalska, M.; Moniuszko-Malinowska, A.; Kozielewicz, D.; Hawro, M.; Rorat, M.; Sikorska, K.; Jaroszewicz, J.; Kowalska, J.; Flisiak, R. Retrospective analysis of the effectiveness of remdesivir in COVID-19 treatment during periods dominated by Delta and Omicron SARS-CoV-2 variants in clinical settings. *J. Clin. Med.* **2023**, *12*, 2371.
52. Flisiak, R.; Zarębska-Michaluk, D.; Rogalska, M.; Kryńska, J.A.; Kowalska, J.; Dutkiewicz, E.; Dobrowolska, K.; Jaroszewicz, J.; Moniuszko-Malinowska, A.; Rorat, M.; Podlasin, R.; Tronina, O.; Rzymski, P. Real-world experience with molnupiravir during the period of SARS-CoV-2 Omicron variant dominance. *Pharmacological Reports* **2022**, *74*, 1279–1285.
53. Schilling, W.H.K.; Jittamala, P.; Watson, J.A.; Boyd, S.; Luvira, V.; Siripoon, T.; Ngamprasertchai, T.; Batty, E.M.; Cruz, C.; Callery, J.J.; Singh, S.; Saroj, M.; Kruabkontho, V.; Ngernseng, T.; Tanglakmankhong, N.; Tubprasert, J.; Abdad, M.Y.; Madmanee, W.; Kouhathong, J.; Suwannasin, K.; Pagornrat, W.; Piaraksa, N.; Hanboonkunupakarn, P.; Hanboonkunupakarn, B.; Poovorawan, K.; Potaporn, M.; Srisubat, A.; Loharjun, B.; Taylor, W.R.J.; Chotivanich, V.; Chotivanich, K.; Imwong, M.; Pukrittayakamee, S.; Dondorp, A.M.; Day, N.P.J.; Teixeira, M.M.; Piyaphanee, W.; Phumratanaprapin, W.; White, N.J. Antiviral efficacy of molnupiravir versus ritonavir-boosted nirmatrelvir in patients with early symptomatic COVID-19 (PLATCOV): an open-label, phase 2, randomized, controlled, adaptive trial. *Lancet Infect. Dis*. **2024**, *24*, 36-45.
54. Xie, Y.; Bowe, B.; Al-Aly, Z. Nirmatrelvir and risk of hospital admission or death in adults with covid-19: emulation of a randomized target trial using electronic health records. *BMJ*. **2023**, *381*, e073312
55. Schwartz, K.L.; Wang, J.; Tadrous, M.; Langford, B.J.; Daneman, N.; Leung, V.; Gomes, T.; Friedman, L.; Daley, P.; Brown, K.A. Population-based evaluation of the effectiveness of nirmatrelvir-ritonavir for reducing hospital admissions and mortality from COVID-19. *CMAJ*. **2023**, *195*: E220-E226.
56. Hammond, J.; Leister-Tebbe, H.; Gardner, A.; Abreu, P.; Bao, W.; Wisemandle, W.; Baniecki, M.L.; Hendrick, V.M.; Damle, B.; Simón-Campos, A.; Pypstra, R.; Rusnak, J.M. Oral Nirmatrelvir for High-Risk, Nonhospitalized Adults with Covid-19. *N. Engl. J. Med*. **2022**, *386*, 1397-1408. doi:10.1056/NEJMoa2118542
57. Goldman, J.D.; Lye, D.C.B.; Hui, D.S.; Marks, K.M.; Bruno, R.; Montejano, R.; Spinner, C.D.; Galli, M.; Ahn, M.Y.; Nahass, R.G.; Chen, Y.S.; SenGupta, D.; Hyland, R.H.; Osinusi, A.O.; Cao, H.; Blair, C.; Wei, X.; Gaggar, A.; Brainard, D.M.; Towner, W.J.; Muñoz, J.; Mullane, K.M.; Marty, F.M.; Tashima, K.T.; Diaz, G.; Subramanian, A. Remdesivir for 5 or 10 days in patients with severe Covid-19. *N. Engl. J. Med*. **2020**, *383*, 1827-1837.
58. Lagevrio - Summary of Product Characteristics, MHRA last updated 19/10/2022 https://www.gov.uk/government/publications/regulatory-approval-of-lagevrio-molnupiravir/summary-of-product-characteristics-for-lagevrio (accessed, 2 march 2025).
59. Paxlovid - Summary of Product Characteristics, EMA last updated 12/11/2024 https://www.ema.europa.eu/en/documents/product-information/paxlovid-epar-product-information_en.pdf (accessed, 2 March 2025).
60. Veklury - Summary of Product Characteristics, EMA last updated 18/12/2024 https://www.ema.europa.eu/en/documents/product-information/veklury-epar-product-information_en.pdf (accessed, 2 March 2025).
61. Liverpool COVID-19 interactions checker, https://www.covid19-druginteractions.org/checker (accessed, 2 March 2025).

62. Ramakrishnan, S.; Nicolau, D.V.; Langford, B.; Mahdi, M.; Jeffers, H.; Mwasuku, C.; Krassowska, K.; Fox, R.; Binnian, I.; Glover, V.; Bright, S.; Butler, C.; Cane, J.L.; Halner, A.; Matthews, P.C.; Donnelly, L.E.; Simpson, J.L.; Baker, J.R.; Fadai, N.T.; Peterson, S.; Bengtsson, T.; Barnes, P.J.; Russell, R.E.K.; Bafadhel, M. Inhaled budesonide in the treatment of early COVID-19 (STOIC): a phase 2, open-label, randomized controlled trial. *Lancet Respir. Med*. **2021**, *9*, 763-772.
63. Hobbs, R.; Gbinigie, O.; Ogburn, E.; Yu, L.M.; van Hecke O.; Dorward, J.; Butler, C.; Saville, B. Inhaled Budesonide for COVID-19 in People at Higher Risk of Complications in the Community The UK National Community Randomi. *Ann. Fam. Med*. **2023**, *21*, 3859.
64. Horby, P.; Lim, W.S.; Emberson, J.R.; Mafham, M.; Bell, J.L.; Linsell, L.; Staplin, N.; Brightling, C.; Ustianowski, A.; Elmahi, E.; Prudon, B.; Green, C.; Felton, T.; Chadwick, D.; Rege, K.; Fegan, C.; Chappell, L.C.: Faust, S.N.; Jaki, T.; Jeffery, K.; Montgomery, A.; Rowan, K.; Juszczak, E.; Baillie, J.K.; Haynes, R.; Landray, M.J. Dexamethasone in Hospitalized Patients with Covid-19. *N. Engl. J. Med*. **2021**, *384*, 693-704.
65. Tang, N.; Bai, H.; Chen, X.; Gong, J.; Li, D.; Sun, Z. Anticoagulant treatment is associated with decreased mortality in severe coronavirus disease 2019 patients with coagulopathy. *J. Thromb. Haemost*. **2020**, *18*, 1094-1099.
66. Ayerbe, L.; Risco, C.; Ayis, S. The association between treatment with heparin and survival in patients with Covid-19. *J. Thromb. Thrombolysis*. **2020**, *50*, 298-301.
67. PRINCIPLE Trial Collaborative Group. Azithromycin for community treatment of suspected COVID-19 in people at increased risk of an adverse clinical course in the UK (PRINCIPLE): a randomised, controlled, open-label, adaptive platform trial. *Lancet* **2021**, *397*: 1063-1074.
68. Matta, M.; Gantzer, L.; Chakvetadze, C.; Moussiegt, A.; De Pontfarcy, A.; Lekens, B.; Diamantis, S. Antibiotic prescription in ambulatory care for COVID-19 patients: a cohort analysis in four European countries. *Eur. J. Clin. Microbiol. Infect. Dis*. **2024**, *43*, 115-119.
69. Hekmat, H.; Rasooli, A.; Siami, Z.; Rutajengwa, K.A.; Vahabi, Z.; Mirzadeh, F.A. A Review of Antibiotic Efficacy in COVID-19 Control. *J. Immunol. Res*. **2023**, *2023*, 6687437.
70. Tomasiewicz, K.; Piekarska, A.; Stempkowska-Rejek, J.; Serafińska, S.; Gawkowska, A.; Parczewski, M.; Niścigorska-Olsen, J.; Łapiński, T.W.; Zarębska-Michaluk, D.; Kowalska, J.D.; Horban, A.; Flisiak, R. Tocilizumab for patients with severe COVID-19: a retrospective, multi-center study. *Expert. Rev. Anti Infect. Ther*. **2021**, *19*, 93-100.
71. Flisiak, R.; Jaroszewicz, J.; Rogalska, M.; Łapiński, T.; Berkan-Kawińska, A.; Bolewska, B.; Tudrujek-Zdunek, M.; Kozielewicz, D.; Rorat, M.; Leszczyński, P.; Kłos, K.; Kowalska, J.; Pabjan, P.; Piekarska, A.; Mozer-Lisewska, I.; Tomasiewicz, K.; Pawłowska, M.; Simon, K.; Polańska, J.; Zarębska-Michaluk, D. Tocilizumab improves the prognosis of COVID-19 in patients with high IL-6. *J. Clin. Med*. **2021**, *10*, 1583.
72. Zarębska-Michaluk, D.; Jaroszewicz, J.; Rogalska, M.; Martonik, D.; Pabjan, P.; Katarzyna Berkan-Kawińska, A.; Bolewska, B.; Oczko-Grzesik, B.; Kozielewicz, D.; Tudrujek-Zdunek, M.; Kowalska, J.D.; Moniuszko-Malinowska, A.; Kłos, K.; Rorat, M.; Leszczyński, P.; Piekarska, A.; Polańska, J.; Flisiak R. Effectiveness of Tocilizumab with and without Dexamethasone in Patients with Severe COVID-19: A Retrospective Study. *J. Inflamm. Res*. **2021**, *14*, 3359-3366.
73. REMAP-CAP Investigators, Gordon AC, Mouncey PR, et al. Interleukin-6 Receptor Antagonists in Critically Ill Patients with Covid-19. *N. Engl. J. Med*. **2021**, *384*, 1491-1502.
74. RECOVERY Collaborative Group. Tocilizumab in patients admitted to hospital with COVID-19 (RECOVERY): a randomised, controlled, open-label, platform trial. *Lancet* **2021**, *397*, 1637-1645.

75. Almskog, L.M.; Sjöström, A.; Sundén-Cullberg, J.; Taxiarchis, A.; Ågren, A.; Freyland, S.; Börjesson, M.; Wikman, A.; Wahlgren, C.M.; Wanecek, M.; van der Linden, J.; Antovic, J.; Lampa, J.; Magnusson, M. Tocilizumab reduces hypercoagulation in COVID-19 - Perspectives from the coagulation and immunomodulation Covid assessment (Coag-ImmCovA) clinical trial. *Thromb. Res*. **2024**, *243*: 109135.
76. RoActemra. Summary of Product Characteristics. https://ec.europa.eu/health/documents/community-register/2011/20110801106350/anx_106350_pl.pdf (accessed, 2 March 2025).
77. Naik, N.B.; Puri, G.D.; Kajal, K.; Mahajan, V. High-dose dexamethasone versus tocilizumab in moderate to severe COVID-19 pneumonia: a randomized controlled trial. *Cureus* **2021**, *13*, e20353.
78. RECOVERY Collaborative Group. Higher dose corticosteroids in patients admitted to hospital with COVID-19 who are hypoxic but not requiring ventilatory support (RECOVERY): a randomised, controlled, open-label, platform trial. *Lancet*. **2023**, *401*, 1499–1507.
79. Wang, S.; Chen, Z.; Zhang, X.; Wu, X.; Wang, Y.; Zhang, Q.; Huang L.; Cui, X.; Cai, Y.; Huang, X.; Xia, J.; Gu, S.; Li, M.; Zhan, Q. Impact of corticosteroid doses on prognosis of severe and critical COVID-19 patients with Omicron variant infection: a propensity score matching study. *Inflammopharmacology*. **2024**, *32*, 3347-3356.
80. Olumiant – Highlights of prescribing information, FDA last updated 16/01/2025 https://www.accessdata.fda.gov/drugsatfda_docs/label/2022/207924s006lbl.pdf (accessed, 2 March 2025).
81. Kalil, A.C.; Patterson, T.F.; Mehta, A.K.; Tomashek, K.M.; Wolfe, C.R.; Ghazaryan, V.; Marconi, V.C.; Ruiz-Palacios, G.M.; Hsieh, L.; Kline, S.; Tapson, V.; Iovine, N.M.; Jain, M.K.; Sweeney, D.A.; El Sahly, H.M.; Branche, A.R.; Pineda, J.R.; Lye, D.C.; Sandkovsky, U.; Luetkemeyer, A.F.: Cohen, S.H.; Finberg, R.W.; Jackson, P.E.H.; Taiwo, B.; Paules, C.I.; Arguinchona, H.; Erdmann, N.; Ahuja, N.; Frank, M.; Oh, M.; Kim, E.S.; Tan, S.Y.; Mularski, R.A.; Nielsen, H.: Ponce, P.O.; Taylor, B.S.; Larson, L.; Rouphael, N.G.: Saklawi, Y.; Cantos, V.D.; Ko, E.R.; Engemann, J.J.: Amin, A.N.: Watanabe, M.; Billings, J.; Elie, M.C.; Davey, R.T.; Burgess, T.H.; Ferreira, J.; Green, M.; Makowski, M.; Cardoso, A.; de Bono, S.; Bonnett, T.; Proschan, M.; Deye, G.A.; Dempsey, W.; Nayak, S.U.; Dodd, L.E.; Beigel, J.H. Baricitinib plus Remdesivir for Hospitalized Adults with Covid-19. *N. Engl. J. Med*. **2021**, *384*, 795-807.
82. Ely, E.W.; Ramanan, A.V.; Kartman, C.E.; de Bono, S.; Liao, R.; Piruzeli, M.L.B.; Goldman, J.D.; Saraiva, J.F.K.; Chakladar, S.; Marconi, V.C. Efficacy and safety of baricitinib plus standard of care for the treatment of critically ill hospitalised adults with COVID-19 on invasive mechanical ventilation or extracorporeal membrane oxygenation: an exploratory, randomised, placebo-controlled trial. *Lancet Respir. Med*. **2022**, *10*, 327-336.
83. Wolfe, C.R.; Tomashek, K.M.; Patterson, T.F.; Gomez, C.A.; Marconi, V.C.; Jain, M.K.; Yang, O.O.; Paules, C.I.; Palacios, G.M.R.; Grossberg, R.; Harkins, M.S.; Mularski, R.A.; Erdmann, N.; Sandkovsky, U.; Almasri, E.; Pineda, J.R.; Dretler, A.W.; de Castilla, D.L.; Branche, A.R.; Park, P.K.; Mehta, A.K.; Short, W.R.; McLellan, S.L.F.; Kline, S.; Iovine, N.M.; El Sahly, H.M.; Doernberg, S.B.; Oh, M.; Huprikar, N.; Hohmann, E.; Kelley, C.F.; Holodniy, M.; Kim, E.S.; Sweeney, D.A.; Finberg, R.W.; Grimes, K.A.; Maves, R.C.; Ko, E.R.; Engemann, J.J.; Taylor, B.S.; Ponce, P.O.; Larson, L.; Melendez, D.P.; Seibert, A.M.; Rouphael, N.G.; Strebe, J.; Clark, J.L.; Julian, K.G.; de Leon, A.P.; Cardoso, A.; de Bono, S.; Atmar, R.L.; Ganesan, A.; Ferreira, J.L.; Green, M.; Makowski, M.; Bonnett, T.; Beresnev, T.; Ghazaryan, V.; Dempsey, W.; Nayak, S.U.; Dodd, L.E.; Beigel, J.H.; Kalil, A.C. Baricitinib versus dexamethasone for adults hospitalised with COVID-19 (ACTT-4): a randomised, double-blind, double placebo-controlled trial. *Lancet Respir. Med.* **2022**, *10*, 888-899.
84. Granholm, A.; Munch, M.W.; Myatra, S.N.; Vijayaraghavan, B.K.T.; Cronhjort, M.; Wahlin, R.R.; Jakob, S.M.; Cioccari, L.; Kjær, M.B.N.: Vesterlund, G.K.; Meyhoff, T.S.; Helleberg, M.; Møller, M.H.; Benfield, T.; Venkatesh,

B.; Hammond, N.E.; Micallef, S.; Bassi, A.; John, O.; Jha, V.; Kristiansen, K.T.; Ulrik, S.; Jørgensen, V.L.; Smitt, M.; Bestle, M.H.; Andreasen, A.S.; Poulsen, L.M.; Rasmussen, B.S.; Brøchner, A.C.; Strøm, T.; Møller, A.; Khan, M.S.; Padmanaban, A.; Divatia, J.V.; Saseedharan, S.; Borawake, K.; Kapadia, F.; Dixit, S.; Chawla, R.; Shukla, U.; Amin, P.; Chew, M.S.; Wamberg, C.A.: Gluud, C.; Lange, T.; Perner, A. Dexamethasone 12 mg versus 6 mg for patients with COVID-19 and severe hypoxaemia: a pre-planned, secondary Bayesian analysis of the COVID STEROID 2 trial. *Intensive Care Med*. **2022**, *48*, 45-55.

85. Grasselli, G.; Calfee, C.S.; Camporota, L.; Poole, D.; Amato, M.B.P.; Antonelli, M.; Arabi, Y.M.; Baroncelli, F.; Beitler, J.R.; Bellani, G.; Bellingan, G.; Blackwood, B.; Bos, L.D.J.; Brochard, L.; Brodie, D.; Burns, K.E.A.; Combes, A.; D'Arrigo, S.; De Backer, D.; Demoule, A.; Einav, S.; Fan, E.; Ferguson, N.D.; Frat, J.P.; Gattinoni, L.; Guérin, C.; Herridge, M.S.; Hodgson, C.; Hough, C.L.; Jaber, S.; Juffermans, N.P.; Karagiannidis, C.; Kesecioglu, J.; Kwizera, A.; Laffey, J.G.; Mancebo, J.; Matthay, M.A.: McAuley, D.F.; Mercat, A.; Meyer, N.J.; Moss, M.; Munshi, L.; Myatra, S.N.; Gong, M.N.; Papazian, L.; Patel, B.K.; Pellegrini, M.; Perner.; Pesenti, A.; Piquilloud, L.; Qiu, H.; Ranieri, M.V.; Riviello, E.; Slutsky, A.S.; Stapleton, R.D.; Summers, C.; Thompson, T.B.; Valente Barbas, C.S.; Villar, J.; Ware, L.B.; Weiss, B.; Zampieri, F.G.; Azoulay, E.; Cecconi, M. ESICM guidelines on acute respiratory distress syndrome: definition, phenotyping and respiratory support strategies. *Intensive Care Med*. **2023**, *49*, 727–759.
86. Badulak, J.; Antonini, M.V.; Stead, C.M.; Shekerdemian, L.; Raman, L.; Paden, M.L.; Agerstrand, C.; Bartlett, R.H.; Barrett, N.; Combes, A.; Lorusso, R.; Mueller, T.; Ogino, M.T.; Peek, G.; Pellegrino, V.; Rabie, A.A.; Salazar, L.; Schmidt, M.; Shekar, K.; MacLaren, G.; Brodie, D. Extracorporeal Membrane Oxygenation for COVID-19: Updated 2021 Guidelines from the Extracorporeal Life Support Organization. *ASAIO J*. **2021**, *67*, 485-495.
87. Goodfellow, L.T.; Miller, A.G.; Varekojis, S.M.; LaVita, C.J.; Glogowski, J.T.; Hess, D.R. AARC Clinical Practice Guideline: Patient-Ventilator Assessment. *Respiratory Care* **2024**, *69*, 1042-1054.
88. Aliyu, B.; Raji, Y.E.; Chee, H.Y.; Wong, M.Y.; Sekawi Z.B. Systematic review and meta-analysis of the efficacy and safety of oseltamivir (Tamiflu) in the treatment of Coronavirus Disease 2019 (COVID-19). *PLoS ONE* **2022**, *17*, e0277206.
89. Weis, N.; Bollerup, S.; Sund, J.D.; Glamann, J.B.; Vinten, C.; Jensen, L.R.; Sejling, C.; Kledal, T.N.; Rosenkilde, M.M. Amantadine for COVID-19 treatment (ACT) study: a randomized, double-blinded, placebo-controlled clinical trial. *Clin. Microbiol. Infect*. **2023**, *29*, 1313-1319.
90. OVERY Collaborative Group. Lopinavir-ritonavir in patients admitted to hospital with COVID-19 (RECOVERY): a randomized, controlled, open-label, platform trial. *Lancet* **2020**, *10259*, 1345-1352.
91. TOGETHER Investigators. Effect of early treatment with ivermectin among patients with COVID-19. *N. Engl. J. Med.* **2022**, *386*, 1721-1731.
92. McCarthy, M.W.; Naggie, S.; Boulware, D.R.; Lindsell, C.J.; Stewart, T.G.; Felker, G.M.; Jayaweera, D.; Sulkowski, M.; Gentile, N.; Bramante, C.; Singh, U.; Dolor, R.J.; Ruiz-Unger, J.; Wilson, S.; DeLong, A.; Remaly, A.; Wilder, R.; Collins, S.; Dunsmore, S.E.; Adam, S.J.; Thicklin, F.; Hanna, G.; Ginde, A.A.; Castro, M.; McTigue, K.; Shenkman, E.; Hernandez A.F. Effect of fluvoxamine vs placebo on time to sustained recovery in outpatients with mild to moderate COVID-19: a randomized clinical trial. *JAMA* **2023**, *329*, 296-305.
93. ITAC Study Group. Hyperimmune immunoglobulin for hospitalised patients with COVID-19 (ITAC): a double-blind, placebo-controlled, Phase 3, randomised trial. *Lancet* **2022**, *399*, 530-540.
94. Francica, J.R.; Cai, Y.; Diallo, S.; Rosenthal, K.; Ren, K.; Flores, D.J.; Dippel, A.; Wu, Y.; Chen, X.; Cantu, E.; Choudhary, R.; Sulikowski, M.; Adissu, H.; Chawla, B.; Kar, S.; van Dyk, N.; Oganesyan, V.; Rajan, S.; Ryan, P.C.; Loo, Y.M.; Cohen, T.; Esser, M.T.; Blair, W. The SARS-CoV-2 Monoclonal Antibody AZD3152 Potently Neutralizes

Historical and Emerging Variants and is Being Developed for the Prevention and Treatment of COVID-19 in High-risk Individuals. *Open Forum Infect. Dis*. **2023**; *10* (Suppl 2), ofad500.1192.
95. RECOVERY Collaborative Group. Convalescent plasma in patients admitted to hospital with COVID-19 (RECOVERY): a randomised controlled, open-label, platform trial. *Lancet* **2021**; *397(10289)*: 2049-2059.
96. Chen, J.S.; Alfajaro, M.M.; Chow, R.D.; Wei, J.; Filler, R.B.; Eisenbarth, S.C.; Wilen, C.B. Non-steroidal anti-inflammatory drugs dampen the cytokine and antibody response to SARS-CoV-2 infection. *J. Virology* **2021**, *95*, e00014-21.
97. Fanlo, P.; Gracia-Tello, B.D.C.; Fonseca Aizpuru, E.; Álvarez-Troncoso, J.; Gonzalez, A.; Prieto-González, S.; Freire, M.; Argibay, A.B.; Pallarés, L.; Todolí, J.A.; Pérez, M.; Buján-Rivas, S.; Ibáñez, B.; GEAS-SEMI Group. Efficacy and Safety of Anakinra Plus Standard of Care for Patients With Severe COVID-19: A Randomized Phase 2/3 Clinical Trial. *JAMA Netw. Open* **2023**, *6*, e237243.
98. Bramante, C.T.; Buse, J.B.; Liebovitz, D.M.; Nicklas, J.M.; Puskarich, M.A.; Cohen, K.; Belani, H.K.; Anderson, B.J.; Huling, J.D.; Tignanelli, C.J.; Thompson, J.L.; Pullen, M.; Wirtz, E.L.; Siegel, L.K.; Proper, J.L.; Odde, D.J.; Klatt, N.R.; Sherwood, N.E.; Lindberg, S.M.; Karger, A.B.; Beckman, K.B.; Erickson, S.M.; Fenno, S.L.; Hartman, K.M.; Rose, M.R.; Mehta, T.; Patel, B.; Griffiths, G.; Bhat, N.S.; Murray, T.A.; Boulware, D.R. COVID-OUT Study Team. Outpatient treatment of COVID-19 and incidence of post-COVID-19 condition over 10 months (COVID-OUT): a multicentre, randomised, quadruple-blind, parallel-group, Phase 3 trial. *Lancet Infect. Dis*. **2023**, *23*, 1119-1129.
99. Thomas, S.; Patel, D.; Bittel, B.; Wolski, K.; Wang, Q.; Kumar, A.; Il'Giovine, Z.J.; Mehra, R.; McWilliams, C.; Nissen, S.E.; Desai, M.Y. Effect of high-dose zinc and ascorbic acid supplementation vs usual care on symptom length and reduction among ambulatory patients with SARS-CoV-2 infection: the COVID A to Z randomized clinical trial. *JAMA Netw. Open*. **2021**, *4*, e210369.
100. Antonazzo, I.C.; Fornari, C.; Rozza, D.; Conti, S.; di Pasquale, R.; Cortesi, P.; Kaleci, S.; Ferrara, P.; Zucchi, A.; Maifredi, G.; Silenzi, A.; Cesana, G.; Mantovani, L.G.; Mazzaglia, G. Azithromycin use and outcomes in patients with COVID-19: an observational real-world study. *Int. J. Infect. Dis*. **2022**, *124*, 27-34.
101. WHO Solidarity Trial Consortium. Repurposed antiviral drugs for Covid-19 - Interim WHO Solidarity trial results. *N. Engl. J. Med*. **2021**, *384*, 497-511.
102. National Institute of Health. COVID-19 Treatment Guidelines. Clinical Spectrum of SARS-CoV-2 Infection. https://www.covid19treatmentguidelines.nih.gov/overview/clinical-spectrum/ (accessed, 2 March 2025)
103. World Health Organization. A clinical case definition of post COVID-19 condition by a Delphi consensus. https://www.who.int/publications/i/item/WHO-2019-nCoV-Post_COVID-19_condition-Clinical_case_definition-2021.1 (accessed, 2 March 2025)
104. Hoshijima, H.; Mihara, T.; Seki, H.; Hyuga, S.; Kuratani, N.; Shiga, T. Incidence of long-term post-acute sequelae of SARS-CoV-2 infection related to pain and other symptoms: A systematic review and meta-analysis. *PLoS One* **2023**, *18*, e0250909.
105. Okarska-Napierała, M.; Ludwikowska, K.; Jackowska, T.; Książyk, J.; Buda, P.; Mazur, A.; Szenborn, L.; Werner, B.; Wysocki, J.; Kuchar, E. Approach to a child with pediatric inflammatory multisystemic syndrome with COVID-19. *Przegl. Pediatr*. **2021**; *50*: 1-11.
106. Niedziela, J.T.; Głowacki, J.; Ochman, M.; Pudlo, R.; Adamczyk-Sowa, M.; Nowowiejska-Wiewióra, A.; Kułaczkowska, Z.; Sobala-Szczygieł, B.; Myrda, K.; Wiewióra, M.; Jaworska, I.; Czapla, K.; Grzanka, A.; Gąsior, M.; Jaroszewicz, J. Post-COVID-19 complications in hospitalized and nonhospitalized patients: the Silesian database of COVID-19 complications (SILCOV-19). *Pol. Arch. Intern. Med*. **2022**, *132*, 16233.

107. Huang. L.W.; Li, H.M.; He, B.; Wang, X.B.; Zhang, Q.Z.; Peng, W.X. Prevalence of cardiovascular symptoms in post-acute COVID-19 syndrome: a meta-analysis. *BMC Med*. **2025**, *23*, 70.
108. Shah, D.P.; Thaweethai, T.; Karlson, E.W.; Bonilla, H.; Horne, B.D.; Mullington, J.M.; Wisnivesky, J.P.; Hornig, M.; Shinnick, D.J.; Klein, J.D.; Erdmann, N.B.; Brosnahan, S.B.; Lee-Iannotti, J.K.; Metz, T.D.; Maughan, C.; Ofotokun, I.; Reeder, H.T.; Stiles, L.E.; Shaukat, A.; Hess, R.; Ashktorab, H.; Bartram, L.; Bassett, I.V.; Becker, J.H.; Brim, H.; Charney, A.W.; Chopra, T.; Clifton, R.G.; Deeks, S.G.; Erlandson, K.M.; Fierer, D.S.; Flaherman, V.J.; Fonseca, V.; Gander, J.C.; Hodder, S.H.; Jacoby, V.L.; Kotini-Shah, P.; Krishnan, J.A.; Kumar, A.; Levy, B.D.; Lieberman, D.; Lin, J.J.; Martin, J.N.; McComsey, G.A.: Moukabary, T.; Okumura, M.J.; Peluso, M.J.; Rosen, C.J.; Saade, G.; Shah, P.K.; Sherif, Z.A.; Taylor, B.S.; Tuttle, K.R. Urdaneta, A.E.; Wallick, J.A.; Wiley, Z.; Zhang, D.; Horwitz, L.I.; Foulkes, A.S.; Singer, N.G. RECOVER Consortium. Sex Differences in Long COVID. *JAMA Netw. Open*. **2025**, *8*, e2455430.
109. Terry, P.; Heidel, R.E.; Wilson, A.Q.; Dhand, R. Risk of long covid in patients with pre-existing chronic respiratory diseases: a systematic review and meta-analysis. *BMJ Open Respir. Res*. **2025**, *12*, e002528.
110. Peter, R.S.; Nieters, A.; Göpel, S.; Merle, U.; Steinacker, J.M.; Deibert, P.; Friedmann-Bette, B.; Nieß, A.; Müller, B.; Schilling, C.; Erz, G.; Giesen, R.; Götz, V.; Keller, K.; Maier, P.; Matits, L.; Parthé, S.; Rehm, M.; Schellenberg, J.; Schempf, U.; Zhu, M.; Kräusslich, H.G.; Rothenbacher, D.; Kern, W.V. EPILOC Phase 2 Study Group. Persistent symptoms and clinical findings in adults with post-acute sequelae of COVID-19/post-COVID-19 syndrome in the second year after acute infection: A population-based, nested case-control study. *PLoS Med*. **2025**, *22*, e1004511.
111. The Royal Australian College of General Practitioners. Caring for patients with post–COVID–19 conditions. December 2021. https://www.racgp.org.au/clinical-resources/covid-19-resources/clinical-care/caring-for-patients-with-post-covid-19-conditions (accessed, 2 March 2025)
112. Spyropoulos, A.C.; Anderson Jr, F.A.; FitzGerald, G.; Decousus, H.; Pini, M.; Chong, B.H.; Zotz, R.B.; Bergmann, J.F.; Tapson, V.; Froehlich, J.B.; Monreal, M.; Merli, G.J.; Pavanello, R.; Turpie, A.G.G.; Nakamura, M.; Piovella, F.; Kakkar, A.K.; Spencer, F.A.; & IMPROVE Investigators. Predictive and associative models to identify hospitalized medical patients at risk for VTE. *Chest* **2011**, *140*, 706-714.
113. Spyropoulos, A.C.; Levy, J.H.; Ageno, W.; Connors, J.M.; Hunt, B.J.; Iba, T.; Levi, M.; Samama, C.M.; Thachil, J.; Giannis, D.; Douketis, J.D.; & Subcommittee on Perioperative, Critical Care Thrombosis, Haemostasis of the Scientific, Standardization Committee of the International Society on Thrombosis and Haemostasis. Scientific and Standardization Committee communication: Clinical guidance on the diagnosis, prevention, and treatment of venous thromboembolism in hospitalized patients with COVID-19. *J. Thromb. Haemost*. **2020**, *18*, 1859-1865.
114. Del Corral, T.; Fabero-Garrido, R.; Plaza-Manzano, G.; Izquierdo-García, J.; López-Sáez, M.; García-García, R.; López-de-Uralde-Villanueva, I. Effect of respiratory rehabilitation on quality of life in individuals with post-COVID-19 symptoms: A randomised controlled trial. *Ann. Phys. Rehabil. Med*. **2025**, *68*, 101920.
115. Combet, E.; Haag, L.; Richardson, J.; Haig, C.E.; Cunningham, Y.; Fraser, H.L.; Brosnahan, N.; Ibbotson, T.; Ormerod, J.; White, C.; McIntosh, E.; O'Donnell, C.A.; Sattar, N.; McConnachie, A.; Lean, M.E.J.; Blane, D.N. Remotely delivered weight management for people with long COVID and overweight: the randomized wait-list-controlled ReDIRECT trial. *Nat. Med.* **2025**, *31*, 258-266.
116. Chow, N.K.N.; Tsang, C.Y.W.; Chan, Y.H.; Telaga, S.A.; Ng, L.Y.A.; Chung, C.M.; Yip, Y.M.; Cheung, P.P. The effect of pre-COVID and post-COVID vaccination on long COVID: A systematic review and meta-analysis. *J. Infect.* **2024**, *89*, 106358.

117. Sun, G.; Lin, K.; Ai, J.; Zhang, W. The efficacy of antivirals, corticosteroids, and monoclonal antibodies as acute COVID-19 treatments in reducing the incidence of long COVID: a systematic review and meta-analysis. *Clin. Microbiol. Infect*. **2024**, *30*, 1505-1513.
118. Oeser, C.; Whitaker, H.; Borrow, R.; Linley, E.; Tonge, S.; Rowe, C.; Otter, A.; Warrener, L.; Campbell, C.; Ladhani, S.; Ramsay, M.; Brown, K.E.; Amirthalingam, G. Following the Omicron wave, the majority of children in England have evidence of previous COVID infection. *J. Infect*. **2023**, *86*, 256–308.
119. Franczak, J.; Moppert, J.; Sobolewska-Pilarczyk, M,; Pawłowska, M. The Seroprevalence of SARS-CoV-2 IgG Antibodies in Children Hospitalized for Reasons Other Than COVID-19. *J. Clin. Med*. **2022**, *11*, 3819. doi: 10.3390/jcm11133819.
120. Luo, C.; Chen, W.; Cai, J.; He, Y. The mechanisms of milder clinical symptoms of COVID-19 in children compared to adults. *Ital. J. Pediatr*. **2024**, *50*, 28.
121. Powell, A.A.; Dowell, A.C.; Moss, P.; Ladhani, S.N.; sKIDs Investigation Team. Current state of COVID-19 in children: 4 years on. *J. Infect*. **2024**, *88*, 106134.
122. Pokorska-Śpiewak, M.; Talarek, E.; Mania, A.; Pawłowska, M.; Popielska, J.; Zawadka, K.; Figlerowicz, M.; Mazur-Melewska, K.; Faltin, K.; Ciechanowski, P.; Łasecka-Zadrożna, J.; Rudnicki, J.; Hasiec, B.; Stani, M.; Frańczak-Chmura, P.; Zaleska, I.; Szenborn, L.; Toczyłowski, K.; Sulik, A.; Szczepańska, B.; Pałyga-Bysiecka, I.; Kucharek, I.; Sybilski, A.; Sobolewska-Pilarczyk, M.; Dryja, U.; Majda-Stanisławska, E.; Niedźwiecka, S.; Kuchar, E.; Kalicki, B.; Gorczyca, A.; Marczyńska, M. Clinical and Epidemiological Characteristics of 1283 Pediatric Patients with Coronavirus Disease 2019 during the First and Second Waves of the Pandemic-Results of the Pediatric Part of a Multicenter Polish Register SARSTer. *J Clin Med* **2021**, *10*, 5098.
123. Pawłowska, M.; Pokorska-Śpiewak, M.; Talarek, E.; Mania, A.; Hasiec, B.; Żwirek-Pytka, E.; Stankiewicz, M.; Stani, M.; Frańczak-Chmura, P.; Szenborn, L.; Zaleska, I.; Chruszcz, J.; Majda-Stanisławska, E.; Dryja, U.; Gąsiorowska, K.; Figlerowicz, M.; Mazur-Melewska, K.; Faltin, K.; Ciechanowski, P.; Peregrym, M.; Łasecka-Zadrożna, J.; Rudnicki, J.; Szczepańska, B.; Pałyga-Bysiecka, I.; Rogowska, E.; Hudobska-Nawrot, D.; Domańska-Granek, K.; Sybilski, A.; Kucharek, I.; Franczak, J.; Sobolewska-Pilarczyk, M.; Kuchar, E.; Wronowski, M.; Paryż, M.; Kalicki, B.; Toczyłowski, K.; Sulik, A.; Niedźwiecka, S.; Flisiak, R.; Marczyńska, M. Clinical Course and Severity of COVID-19 in 940 Infants with and without Comorbidities Hospitalized in 2020 and 2021: The Results of the National Multicenter Database SARSTer-PED. *J. Clin. Med*. **2023**, *12*, 2479.
124. Cohen, J.M.; Carter, M.J.; Cheung R.C.; Ladhani, S. Lower risk of multisystem inflammatory syndrome in children (MIS-C) with the Delta and Omicron variants of SARS-CoV-2. *Clin. Infect. Dis*. **2022**, *76*, e518–21.
125. Aparicio, C.; Willis, Z.I.; Nakamura, M.M.; Wolf, J.; Little, C.; Maron, G.M.; Sue, P.K.; Anosike, B.I.; Miller, C.; Bio, L.L.; Singh, P.; James, S.H.; Oliveira, C.R. Risk Factors for Pediatric Critical COVID-19: A Systematic Review and Meta-Analysis. *J. Ped. Infect. Dis. Soc*. **2024**, *13*, 352–362.
126. Mańdziuk, J.; Kuchar, E.; Okarska-Napierała, M. How international guidelines recommend treating children who have severe COVID-19 or risk disease progression. *Acta Paediatr*. **2024**, *113*: 2345-2353.
127. Camporesi, A.; Morello, R.; La Rocca, A.; Zampino, G.; Vezzulli, F.; Munblit, D.; Raffaelli, F.; Valentini, P.; Buonsenso, D. Characteristics and predictors of Long Covid in children: a 3-year prospective cohort study, *eClinicalMedicine* **2024**, *76*, 102815.
128. Orban, E.; Li, L.Y.; Gilbert, M.; Napp, A.K.; Kaman, A.; Topf, S.; Boecker, M.; Devine, J.; Reiß, F.; Wendel, F.; Jung-Sievers, C.; Ernst, V.S.; Franze, M.; Möhler, E.; Breitinger, E.; Bender, S.; Ravens-Sieberer, U. Mental health and

quality of life in children and adolescents during the COVID-19 pandemic: a systematic review of longitudinal studies. *Front. Public Health*. **2024**; *11*: 1275917.
129. Iacopetta, D.; Catalano, A.; Ceramella, J.; Pellegrino, M.; Marra, M.; Scali, E.; Sinicropi, M.S.; Aquaro, S. The Ongoing Impact of COVID-19 on Pediatric Obesity. *Pediatr. Rep*. **2024**, *16*, 135-150.
130. European Medicines Agency. https://www.ema.europa.eu/en/news/etf-recommends-updating-covid-19-vaccines-target-new-jn1-variant (accessed, 2 March 2025)
131. Centers for Disease Control and Prevention. https://www.cdc.gov/vaccines/covid-19/clinical-considerations/covid-19-vaccines-us.html (accessed, 2 March 2025)
132. CDC Interim Clinical Considerations for Use of COVID-19 Vaccines in the United States. https://www.cdc.gov/vaccines/covid-19/clinical-considerations/interim-considerations-us.html# (accessed, 2 March 2025)
133. World Health Organization (WHO) Coronavirus disease (COVID-19) Epidemiological Updates and Monthly Operational Updates. WHO: COVID-19 epidemiological updates. https://www.who.int/emergencies/diseases/novel-coronavirus-2019/situation-reports (accessed, 2 March 2025)
134. Comirnaty. Summary of Product Characteristics. https://www.ema.europa.eu/en/documents/product-information/comirnaty-epar-product-information_en.pdf (accessed, 2 March 2025).
135. Spikevax. Summary of Product Characteristics. https://www.ema.europa.eu/en/documents/product-information/spikevax-epar-product-information_en.pdf (accessed, 2 March 2025).
136. Nuvaxovid. Summary of Product Characteristics. https://www.ema.europa.eu/en/documents/product-information/nuvaxovid-epar-product-information_en.pdf (accessed, 2 March 2025).
137. Ministerstwo Zdrowia – Komunikat Ministra Zdrowia nr 36 z dnia 22 października 2024 r. https://www.gov.pl/attachment/ba7d5589-e7bf-484c-9552-9fe416b5c885 (accessed, 2 march 2025)
138. Ministerstwo Zdrowia Komunikat nr 37 Ministra Zdrowia z 26.11.2024 r. w sprawie realizacji szczepień u dzieci 6 mies.–11 lat dostęp 12 luty 2025. https://www.gov.pl/attachment/801f5af3-f7d1-42ff-857f-d2b868911ff0 (accessed, 2 march 2025)
139. Program Szczepień Ochronnych na rok 2025. https://www.gov.pl/attachment/83bef042-106b-4228-a011-56bcb58470bb (accessed, 2 March 2025)
140. Comirnaty. Periodic safety update report assessment 19 June 2023 to 18 December 2023. www.ema.europa.eu/en/documents/covid-19-vaccine-safety-update/comirnaty-periodic-safety-update-report-assessment-19-june-2023-18-december-2023_en.pdf (accessed, 2 march 2025)
141. Spikevax. Periodic safety update report assessment 18 June 2023 to 17 December 2023. www.ema.europa.eu/en/documents/covid-19-vaccine-safety-update/spikevax-periodic-safety-update-report-assessment-18-june-2023-17-december-2023_en.pdf (accessed, 2 march 2025).
142. WHO. Statement - update on COVID-19: Omicron wave threatening to overcome health workforce, 11-01-2022. https://www.who.int/poland/multi-media/details/statement---update-on-covid-19--omicron-wave-threatening-to-overcome-health-workforce--11-01-2022 (accessed, 2 March 2025).
143. ETF statement on the loss of activity of anti-spike protein monoclonal antibodies due to emerging SARS-CoV-2 variants, 9 December 2024. https://www.ema.europa.eu/en/documents/other/etf-statement-loss-activity-anti-spike-protein-monoclonal-antibodies-due-emerging-sars-cov-2-variants-december-2024-update_en.pdf (accessed, 2 march 2025).
144. Wang, Q.; Guo, Y.; Ho, J.; Ho, D.D. Activity of research-grade pemivibart against recent SARS-CoV-2 JN.1 sublineages. *N. Engl. J. Med*. **2024**, *391*, 1863-1864.

145. Emergency use authorization (EUA) for pemgarda, Center for Drug Evaluation and Research (CDER). Review memorandum. https://www.fda.gov/media/182220/download?attachment (accessed, 2 March 2025)
146. Esmaeili, S.; Owens, K.; Wagoner, J.; Polyak S.J.; White J.M.; Schiffer J.T. A unifying model to explain frequent SARS-CoV-2 rebound after nirmatrelvir treatment and limited prophylactic efficacy. *Nature Commun.* **2024**, *15*, 5478.
147. Rahmati, M., Shamsi, M. M., Khoramipour, K., Malakoutinia, F., Woo, W., Park, S., Yon, D. K., Lee, S. W., Shin, J. I., Smith, L. Baseline physical activity is associated with reduced mortality and disease outcomes in COVID-19: A systematic review and meta-analysis. *Rev. Med. Virol*. **2022**; *32*: e2349.

Disclaimer/Publisher's Note: The statements, opinions and data contained in all publications are solely those of the individual author(s) and contributor(s) and not of MDPI and/or the editor(s). MDPI and/or the editor(s) disclaim responsibility for any injury to people or property resulting from any ideas, methods, instructions or products referred to in the content.
